# Advances and Applications of Micro- and Mesofluidic Systems

**DOI:** 10.1021/acsomega.4c10999

**Published:** 2025-03-25

**Authors:** Larissa Araújo Oliveira Alves, John Hebert da Silva Felix, Antônio Átila Menezes Ferreira, Maria Tayane Barroso dos Santos, Carlos Galvão da Silva, Larysse Maria Santiago de Castro, José Cleiton Sousa dos Santos

**Affiliations:** Instituto de Engenharias e Desenvolvimento Sustentável, Universidade da Integração Internacional da Lusofonia Afro-Brasileira, Campus Auroras, Redenção CEP 62790-970, CE, Brazil

## Abstract

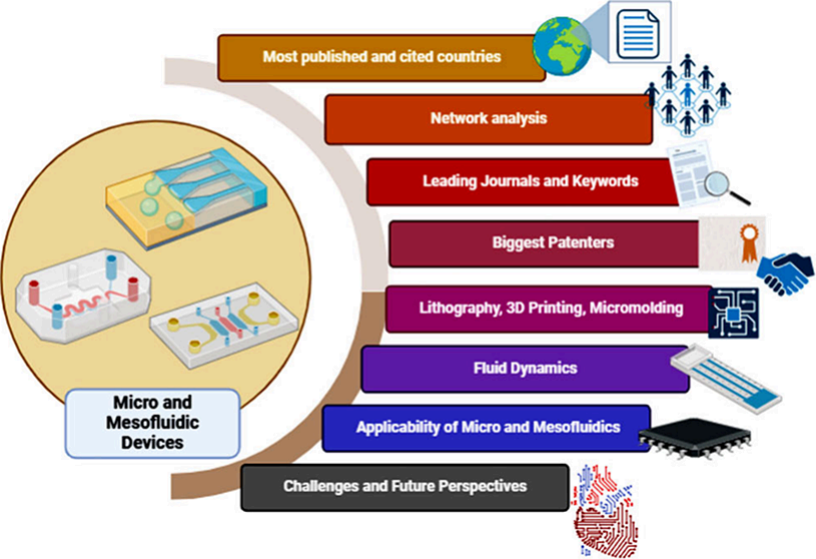

Microfabrication technology has advanced scientific understanding and expanded our molecular control capabilities, enabling the development of 3D models in micrometer structures. The sizes of the fluidic channels are arranged in descending order, starting with the macro-, followed by the meso-, micro-, and nanoscale. These advances bring advantages and speed up biological and chemical experimental processes. Such miniaturized systems show significant advances, particularly in meso- and microreactors, through high-throughput screening. This work proposes a bibliometric analysis of the advances and applications of the Web of Science (WoS) database, analyzing the main highlights of the publications, indicators, and impact on knowledge production. In the past 20 years, approximately 3,934 documents published and cited, mainly by major world powers on micro- and mesofluidic systems, are increasingly expanding in the academic and industrial sectors.

## 1 Introduction

The 1980s were marked by a historic milestone in science: the birth of microfluidic chips. Several renowned researchers, such as George Whitesides (Harvard University), Stephen Quake (Stanford University), and Andreas Manz (University of Twente, Netherlands), have joined forces to bring this innovative technology to life.^1^ Microfluidics has experienced an accelerated development in recent decades, driven by the remarkable innovation of the “micro total analysis system”.^2^

With a field dedicated to miniaturizing fluids on small scales, providing parallel and fast experiments with low reagent consumption.^3^ Microfluidics have many uses and can make complex tasks quite efficient.^4^ In short, microfluidic techniques are practical tools for manipulating and detecting articles or cells.^5^

In this context, the technology has been “shrinking in size”, which was previously limited to the macroscale (>1 mm), and has started to develop in meso (100 μm to 1 mm), micro (100 nm to 100 μm) and nanoscale (<100 nm).^6^ As a result, implementing the technology at a “micro” scale has brought some innovations, including the development of microelectromechanical systems, such as lab-on-a-chip (LOC), which can automate biological and chemical laboratory processes.^7^

The evolution of microfluidics represents a revolution in science and technology, as it allows for the precise manipulation of fluids at microscopic scales. Focusing on the behavior of fluids in systems with small dimensions deviates from the conventional flow theory. Its development brought several devices, such as flow sensors, micropumps, and microvalves, and expanded to other areas, exploring unique phenomena and obtaining applications in areas such as chemical analysis.^8^ Mesofluidic systems are similar to microfluidic systems, except they have a higher flow rate and operate with larger fluid volumes.^9^

The miniaturized flow system for analysis is designed to integrate all the components of a flow collector into one unit, about the size of a credit card. This would imply that instead of the individual manifold components (injection unit, mixing coils, separation units, and flow detectors) being joined together using flexible pipes and nuts. The entire channel system was to be manufactured on a flat surface of a plate, the plate being thick enough to be mechanically stable and to accommodate optical flow cells, electrochemical sensor electrodes, packaged reactors, separation microcolumns, as well as inlets and outlets for the conduits to be connected with external sources of liquids.^10^

Several advantages and disadvantages are associated with micros and mesoreactors. The main advantages of microreactors (10 to 300 mm) are low material consumption, low waste production, excellent mass transfer properties, and rapid diffusive mixing, with disadvantages including low throughput, the tendency to suffer from channel blockage and high-pressure drops. For microreactors (300 mm to more than 5 mm), advantages include high throughput, low-pressure drops, and the ability to handle solids for heterogeneous catalysis. The few disadvantages of mesoreactors are the poorer mass transfer and the slower mixing.^11^

Therefore, the present study aims to perform an advanced bibliometric review of the development and applications of micro and mesofluidic systems.^12−18^ The study uses the Web of Science (WoS) database to analyze the leading publications, indicators, and impacts on knowledge production in micro and mesofluidic data over the last 20 years. The bibliometric analysis seeks to answer questions about the authors, institutions, most relevant countries, main works cited, and topics addressed in the literature and identify emerging trends and future investigations.^19−22^ As a result, the analysis of the documents used in the Web of Science (WoS) database returned 3,934 articles.

## 2 Methodology

This analysis is based on previous research^23−29^ to perform an advanced bibliometric review, a quantitative analysis of scientific production micro/mesofluidic systems research. The database used for the study was the Web of Science (WoS).^30^ The choice to use a single database is justified by the need to ensure the standardization and reliability of the data collected, minimizing the possibility of duplications resulting from using different sources of information.

**Figure 1:**
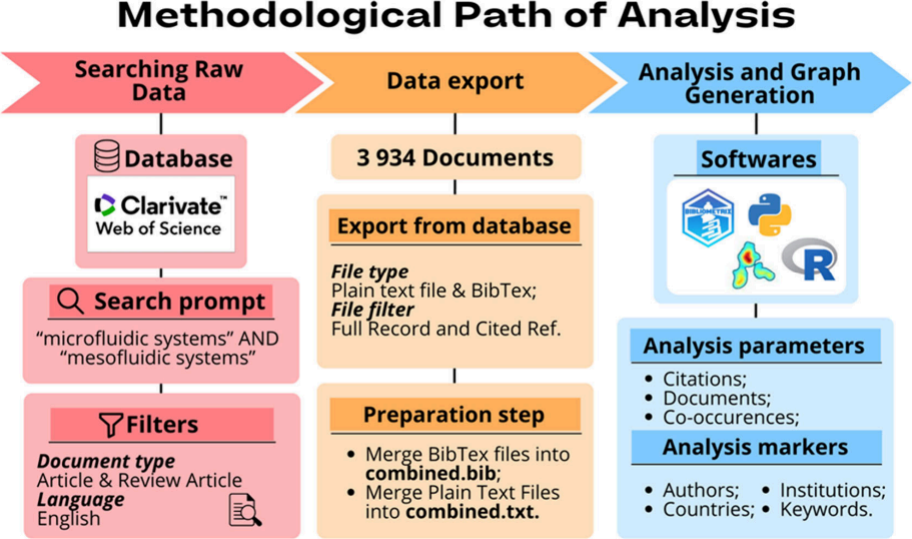
Representation of the search criteria of the study’s methodology and refinement condition in the WoS database.

An advanced search was carried out in the specified database to obtain the most relevant publications, where the keywords “Microfluidic system” and “Mesofluidic system” were combined. The documents were filtered as ‘articles’ and ‘review articles’ without specifying the period. Only documents in English were requested. This search returned 3,934 publications covering the period from 1997 to 2024, evidencing the growth and evolution of research in micro and mesofluidic systems in the last 27 years. Figure 1 shows the methodological path used to research and analyze these data.

The results obtained through the database seek to answer the following questions: Which authors, institutions, and countries are most relevant in this area of research? What are the main works in terms of citation and impact indexes? What is being addressed in the literature on this topic? What research is currently being developed? What future research will be carried out in this field?^31−42^

The bibliometric analysis used the VOSviewer software (version 1.6.20) (https://www.vosviewer.com/), a program used to build and visualize bibliometric networks and Bibliometrics^43^ through the Biblioshiny interface (version 4.1.3), which provides a more comprehensive bibliometric analysis allowing the import of different databases, such as SCOPUS, Web of Science, among others.

## 3 Results

### 3.1 Bibliometric Analysis of the Development and Application of Micro and Mesofluidic Systems

**Figure 2:**
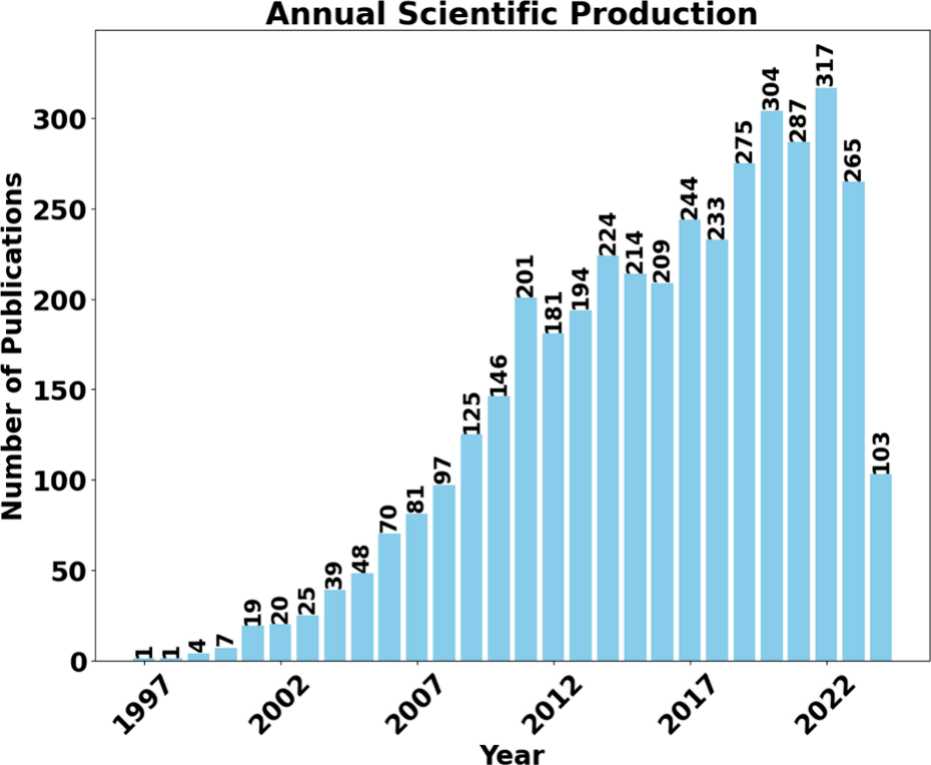
Temporal evolution of publications on micro and mesofluidic systems.

The initial search in the Web of Science (WoS) database resulted in 3,934 articles, of which 3,737 were original research articles and 197 review articles were published between 1997 and 2024, as shown in Figure 2. This analysis of the central database facilitated the identification of the most relevant and cited articles, the most influential authors, institutions, journals, and countries, and the chronological distribution of publications. The most cited article in the research area was “Droplet Microfluidics”, with 2,075 citations. It reviews the droplet microfluidics area comprehensively, highlighting the operations developed for droplet manipulation and its applications in biomedical engineering. It discusses the dimensional advantages of microfluidic droplet systems and the ability to make operations programmable and reconfigurable. It also highlights the diverse applications of these systems in biomedical research and practical applications.^44^

Research showed a significant growth from 2001 onward, with 19 articles published. Figure 2 shows the chronological evolution of publications in the last 15 years. The highest peak in publications took place in 2022, with 317 articles published, with the most relevant topic being “Wearable plasmonic paper-based microfluidics for continuous sweat analysis”, with 105 citations, where it addresses the development of an innovative sensor that combines microfluidics on paper with plasmonic sensors for continuous sweat analysis. This device allows simultaneous quantitative analysis of sweat loss, sweat rate, and metabolism, providing valuable information to monitor health status and disease in individuals. The technology employed in the study has the potential to provide clinically relevant information associated with users’ health and disease states.^45^

In 2023, 265 articles were published, with the most cited being “An overview of the surface with controllable wettability for microfluidic applications” 20 citations, and advocates that surfaces with controllable wettability are used in several areas, including micro and mesofluidic, due to such surfaces play critical roles in processes such as self-cleaning, oil–water separation, and water harvesting. The article reviews the performance of these surfaces as advances and challenges in the modulation of wettability.^46^

In 2024, until June, the date of the data collected in the database, 103 articles were published in micro and mesofluidics, as shown in Figure 2, the temporal development of publications.

#### 3.1.1 Analysis of the Leading Countries

**Figure 3:**
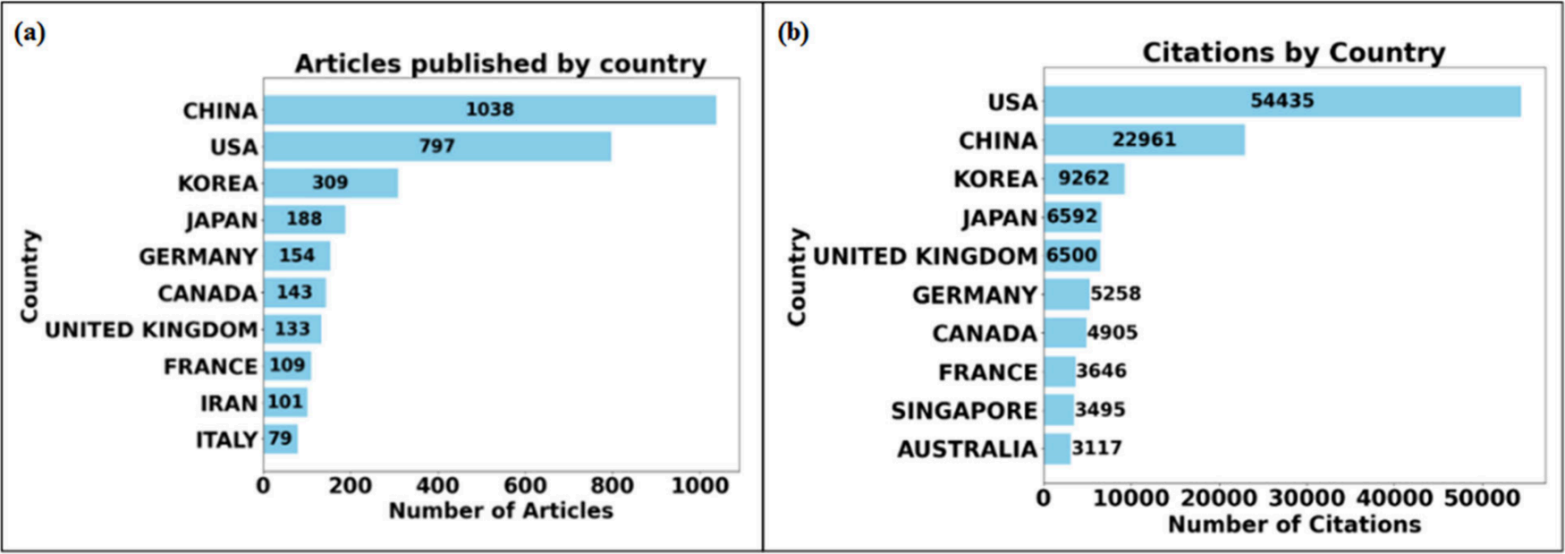
Leading countries that published on micro and mesofluidic systems. (a) The top ten countries that published the most. (b) The top ten most cited countries.

Figure 3 provides information that makes it possible to understand the analysis of the index of publications of articles and citations. Figure 3–(a) presents the ranking of the top ten countries that published the most. Meanwhile, Figure 3–(b) shows the ranking of the most cited countries.

Generally, it is possible to observe that China leads in publications but does not maintain the leadership in citations. The USA ranks second in publications and leads with 54,435 citations. Citations are highly relevant, allowing communities to share results and broaden discussions through research.^47^

#### 3.1.2 Contribution by Institution, Authors, and Co-authors

**Figure 4:**
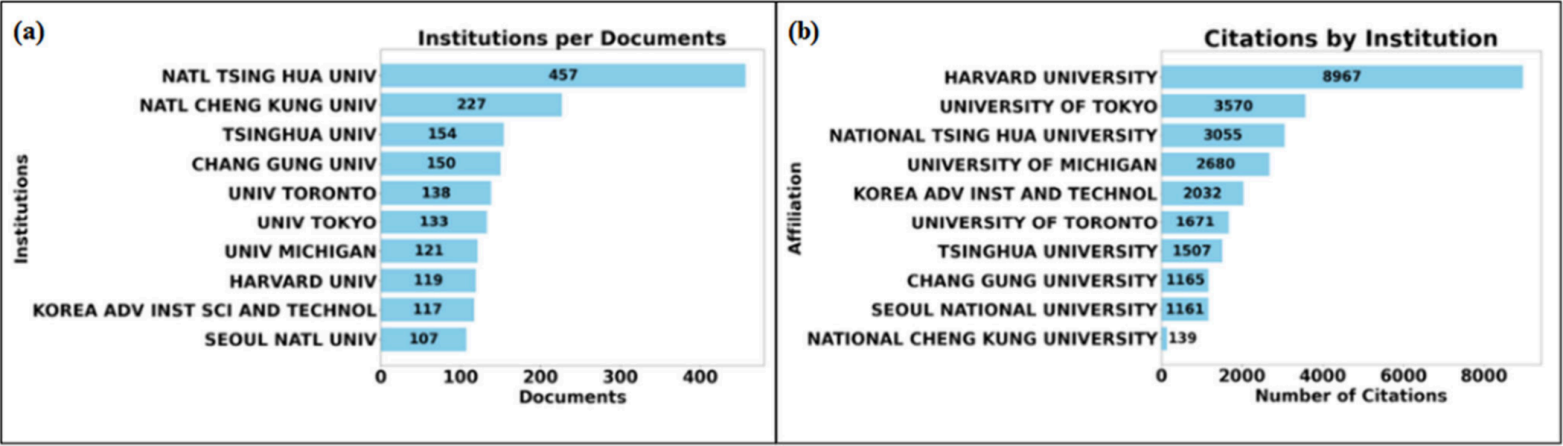
Top ten institutions that published on micro and mesofluidic systems. (a) Institutions that published the most. (b) Most cited institutions.

Figure 4 presents information on the number of publications and citations of the top ten journals that were cited the most. Figure 4–(a) shows the top ten institutions that published the most. Figure 4–(b) shows the top ten institutions that were most cited. It is possible to observe that the institution that leads in publications does not lead in citations. The most cited institution is Harvard University, with 8,967 citations, followed by the University of Tokyo, with 3,570 citations, and National Tsing Hua University, with 3,055 citations.

**Figure 5:**
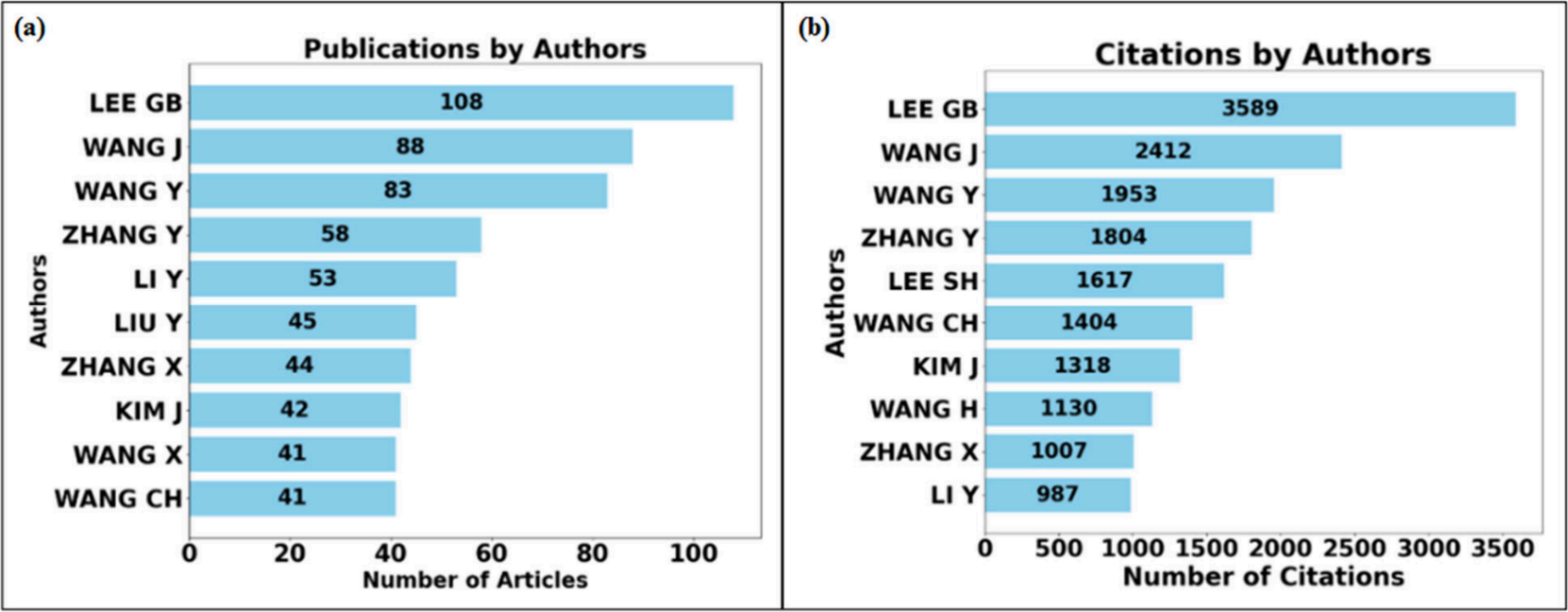
Top ten authors who published on micro and mesofluidic systems. (a) Authors who published the most. (b) Most cited authors.

In more detail, Figure 5 shows the rankings regarding publications and citations. Figure 5–(a) shows the top ten authors who published the most. The author who published the most was Lee GB with 108 publications, followed by Wang J with 88 publications, and Wang Y in third with 83 publications. Figure 5–(b) shows the top ten authors regarding citations. The most cited author was Lee GB, who had 3,589 citations. The second most cited was Ismagilov RF with 3,149 citations, and the third was Ingber DE with 2,957 citations. Regarding the CA index, the author Wang CH has the highest index (CA = 34.24), followed by Lee GB (CA = 33.23), who is the leader in publications and citations.

**Figure 6:**
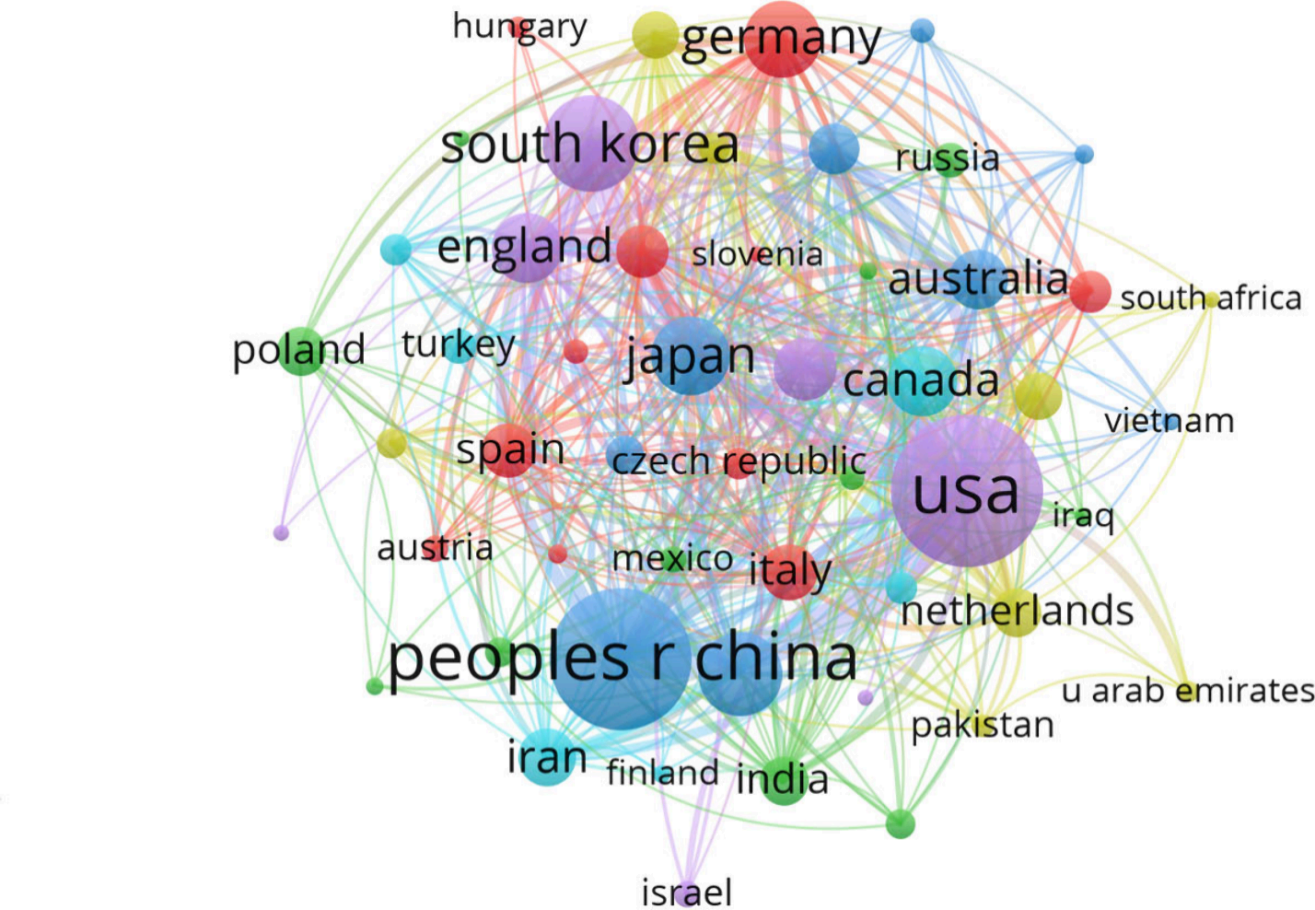
Network map of coauthorship among countries.

Figure 6 presents the analysis of coauthorship among countries. Publications from 52 countries, with a minimum of 5 documents published and five citations, were selected and analyzed using the VOSviewer software tool, resulting in a network map. Three major groups are visible: purple with USA highlighted, blue with China, and purple with South Korea. It can be concluded that the connection between the groups shows citation strengths, which are measured according to citation relationships. The map shows the distribution of countries through collaborations divided into 6 clusters. The total link strength (TLS) index was: the United States with TLS = 466, China with TLS = 304, and South Korea with TLS = 167.

**Figure 7:**
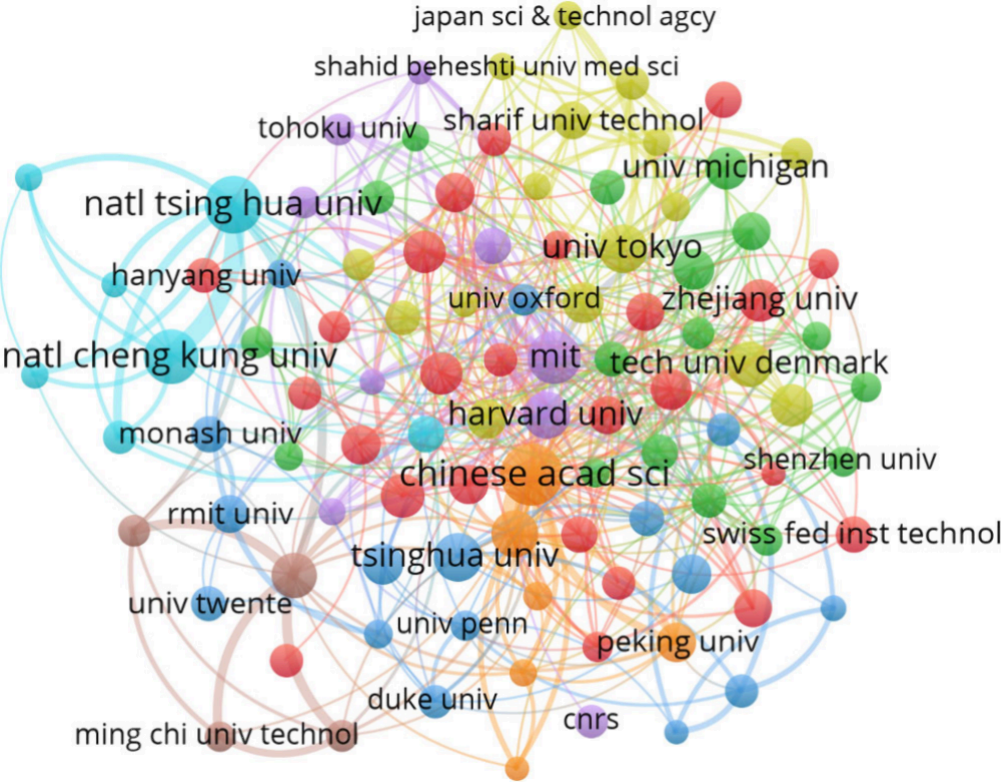
Network map of coauthorship among the top 100 institutions that participated in collaboration.

Regarding the coauthorship analysis, Figure 7 presents the network map where it is possible to visualize collaboration through the parameter to analyze institutions with a minimum of 5 publications and five citations. The analysis identified the cooperation of 376 institutions in the form of coauthorship. The map shows the visualization of the top 100 institutions that participated in collaboration, where three groups stand out: in orange, the Chinese Academy of Sciences; in blue, National Tsing Hua University; and in blue, National Cheng Kung University. This concludes that investigations on micro and mesofluidics are gaining ground and becoming increasingly relevant.

**Figure 8:**
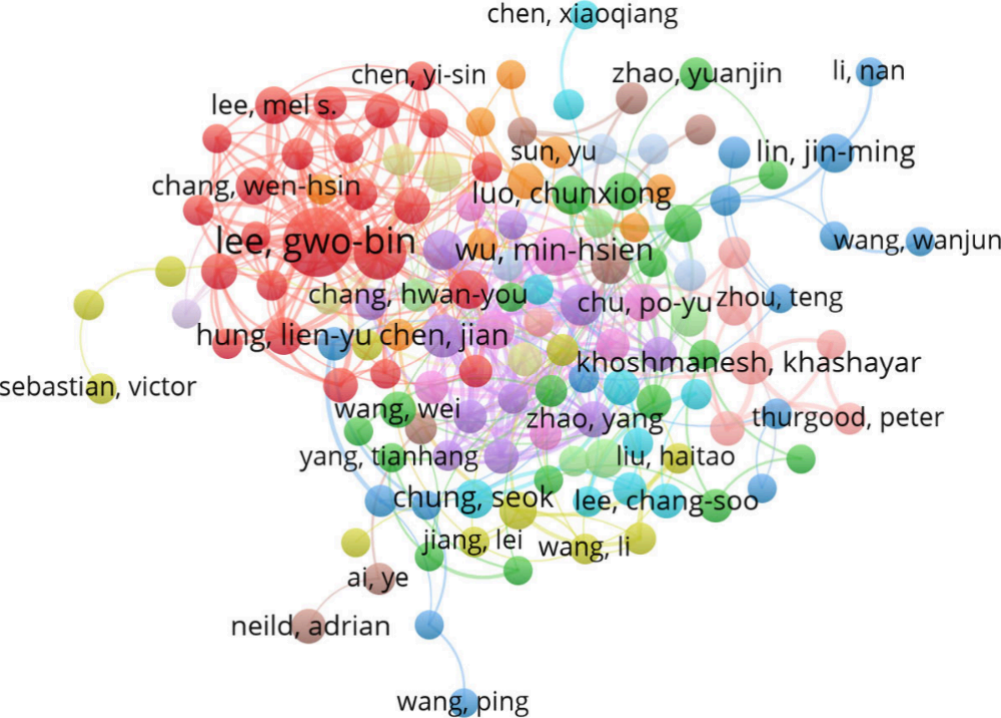
Network map of coauthorship among the 133 authors out of 277 who collaborated.

Figure 8 shows the network map of coauthorship among authors with a minimum of 5 publications and five citations. The analysis identified 277 authors who participated in coauthorship, where the map shows the distribution of the top 133 divided into 14 clusters. The most prominent coauthor, highlighted in red, is Gwo-Bin Lee, according to the analysis parameters.

#### 3.1.3 Distribution of Scientific Journals

**Figure 9:**
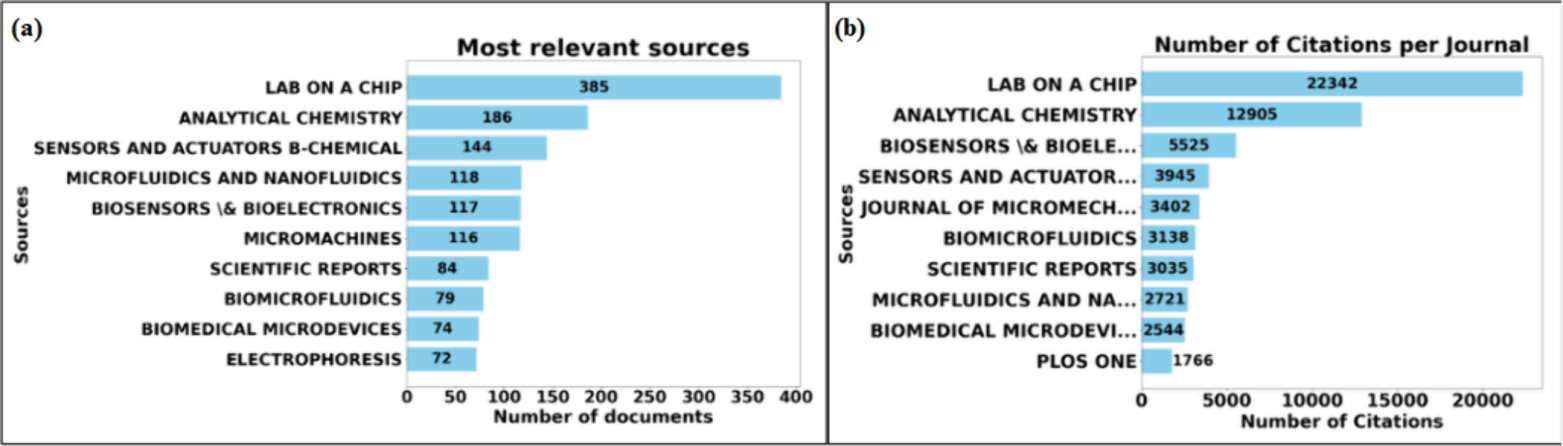
Top journals that published on micro and mesofluidic systems. (a) Top ten journals that published the most. (b) Top ten most cited journals.

Based on the filters established for the database search in this study, 832 journals were identified that were published on micro and mesofluidic systems. Figure 9 provides more information about the journals that published the most. Figure 9–(a) shows the top ten journals that published the most on micro and mesofluidic systems. The journal Lab on a Chip ranks first with 385 publications, Analytical Chemistry with 186 publications, and Sensors and Actuators B: Chemical with 144 publications. Figure 9–(b) shows the top ten most cited journals.

#### 3.1.4 Most Cited Articles in the Research Area of Micro and Mesofluidic Systems

Table 1 ranks the ten most cited scientific articles on micro and mesofluidic systems. The ten articles presented received a total of 12,744 citations. The most cited article was “Droplet microfluidics”, presented in topic 3.1. The second most cited article, with 1,631 citations, was “Dynamic Pattern Formation in a Vesicle-Generating Microfluidic Device”, which aimed to investigate the dynamic formation of patterns in a vesicle-generating microfluidic device. It explores the self-organization of droplets into complex and coherent patterns, demonstrating how the interaction between two immiscible fluids can introduce nonlinearity and instability into the microfluidic device. This phenomenon of complex pattern formation is an example of self-organization in a dynamical system far from thermodynamic equilibrium.^48^

**Table 1:** Ranking of the Top 10 Micro and Mesofluidic Systems Articles

Classification	Title	Author	Year	NC^a^	Reference
1	Droplet microfluidics	Teh S., et al.	2008	2.075	(44)
2	Dynamic Pattern Formation in a Vesicle-Generating Microfluidic Device	Thorsen T., et al.	2001	1.631	(48)
3	Micromixers—a review	Nguyen, N.-T., and Wu, Z.	2005	1.396	(8)
4	From 3D cell culture to organs-on-chips	Huh, et al.	2011	1.298	(49)
5	TEER Measurement Techniques for In Vitro Barrier Model Systems	Srinivasan, et al.	2015	1.273	(50)
6	Human gut-on-a-chip inhabited by microbial flora that experiences intestinal peristalsis-like motions and flow	Kim, et al.	2012	1.119	(51)
7	A Microfluidic System for Controlling Reaction Networks in Time	Song, et al.	2003	1.012	(52)
8	Single-cell analysis and sorting using droplet-based microfluidics	Mazutis, et al.	2013	975	(53)
9	Review Article—Dielectrophoresis: Status of the theory, technology, and applications	Pethig, et al.	2010	965	(54)
10	Micro Total Analysis Systems. Recent Developments	Vilkner, et al.	2004	897	(55)

a NC = Number of citations.

The third most cited article was “Micromixers—a review”, and it analyzes the progress in the recent development of micromixers, addressing advances in micromixer technology, including passive and active designs, and discusses different types of micromixers, their applications, and benefits. The study also highlights the importance of micromixers in several areas, such as chemical analysis, biotechnology, and microfluidics, and explores the most recent trends and innovations in this field.^8^ The fourth most cited article was “From 3D cell culture to organs-on-chips”, which addresses advances in 3D cell culture models, from organs-on-chips to tissue differentiation and disease modeling. It explores how these innovative technologies are revolutionizing the study of human physiology and drug development.^49^

The fifth most cited article, “TEER Measurement Techniques for In Vitro Barrier Model Systems” is a review that discusses the importance of endothelial and epithelial cells in maintaining barrier integrity in the body and explores the use of microengineered devices to study organ function. The study addresses topics such as the composition of the cell culture medium, the spontaneous differentiation of Caco-2 cells, the influence of the cell culture period, and the use of microchip organ models to study the permeability of therapeutic drugs across physiological barriers.^50^ The sixth paper, “Human gut-on-a-chip inhabited by microbial flora that experiences intestinal peristalsis-like motions and flow”, presents the development of an innovative model called “gut-on-a-chip” that replicates the mechanical, structural, and physiological properties of the human gut along with its microbial flora. This microfluidic device has the potential to accelerate pharmaceutical development and reduce the need for animal testing. It provides a controlled environment to study critical intestinal functions in the presence of relevant physiological stimuli, such as cyclic mechanical tension, fluid flow, and the coexistence of microbial flora.^51^

The seventh paper, “A Microfluidic System for Controlling Reaction Networks in Time”, shows a microfluidic system’s development to control network reactions in time. This system allows the formation of aqueous droplets in a microfluidic channel in a continuous flow of an immiscible fluid in water. It acts as microreactors that rapidly mix and transport the reactants without dispersion. These droplets can be used to control networks of chemical reactions on a millisecond time scale, providing precise control over the initiation, evolution, and analysis of responses.^52^ The eighth article, “Single-cell analysis and sorting using droplet-based microfluidics”, deals with droplet-based microfluidic systems for high-speed analysis and separation of individual cells. It outlines a detailed protocol for performing single-cell analyses efficiently and accurately, highlighting the advantages of these systems for cell biology and genomics studies. In addition, the article addresses droplet manipulation, reagent addition, assay measurement, and fluorescence detection for analysis.^53^

The ninth article, “Review Article—Dielectrophoresis: Status of the Theory, technology, and Applications”, presents the current state of the theory, technology, and dielectrophoresis (DEP) applications. The study reviews the progress made in recent years, with about 2000 publications addressing these three aspects. Currently, the trend indicates that theory and technology have matured enough that most efforts are directed toward the application of DEP to unmet needs in areas such as biosensors, cell therapeutics, drug discovery, medical diagnostics, microfluidics, nanoassembly, and particle filtration. The article highlights theoretical advances, including multipolar contributions in describing the DEP force and technological developments, such as new electrode designs and materials used in DEP.^54^

The tenth article, “Micro Total Analysis Systems. Recent Developments”, deals with recent developments in Micro Total Analysis Systems (μTAS), also known as “lab on a chip” or miniaturized analysis systems. It covers a wide range of topics, including microfabrication technologies, bonding techniques, surface modification, design, standard analytical operations, sample preparation, fluid and particle manipulation, reactors, separation, detection, applications in areas such as cell culture, clinical diagnostics, environmental concerns, immunoassays, proteins, DNA analysis, and separation, among others.^55^

#### 3.1.5 Research Areas

**Figure 10:**
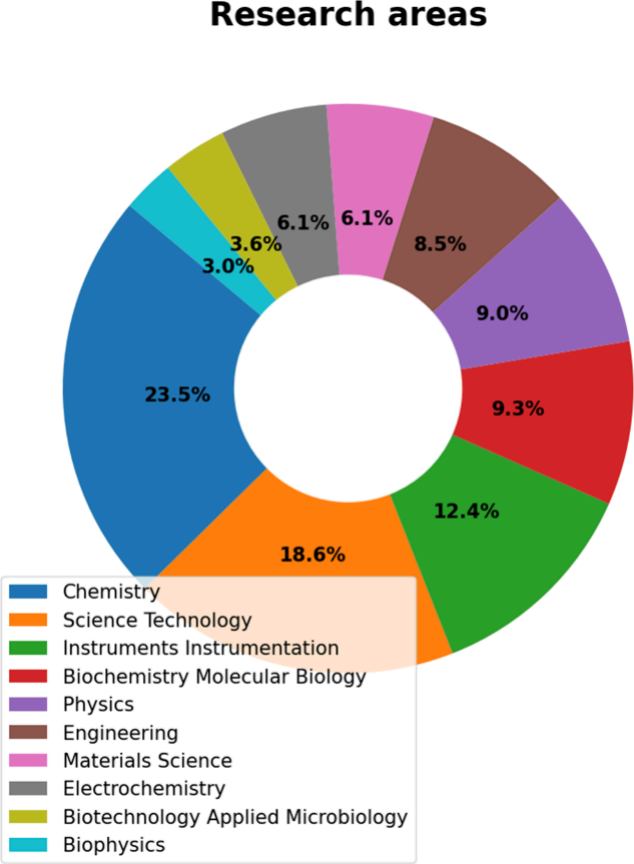
Distribution of the leading research areas of micro and mesofluidic systems articles.

The search in the WoS database resulted in 3,936 documents and identified 90 research areas. It should be noted that an article can fit into multiple research areas. In Figure 10, it is possible to observe the top ten research areas in micro and mesofluidics regarding publications. The percentages were normalized for the main topics, with the field of chemistry standing out with 23.5% of the publications. The second most relevant research area was scientific technology, which was used in 18.6% of the publications. Instrumentation was the third most cited area, with 12.4% of the publications. The most cited article in the top three regions was “Droplet Microfluidics”.^44^

### 3.2 Keyword Analysis

**Figure 11:**
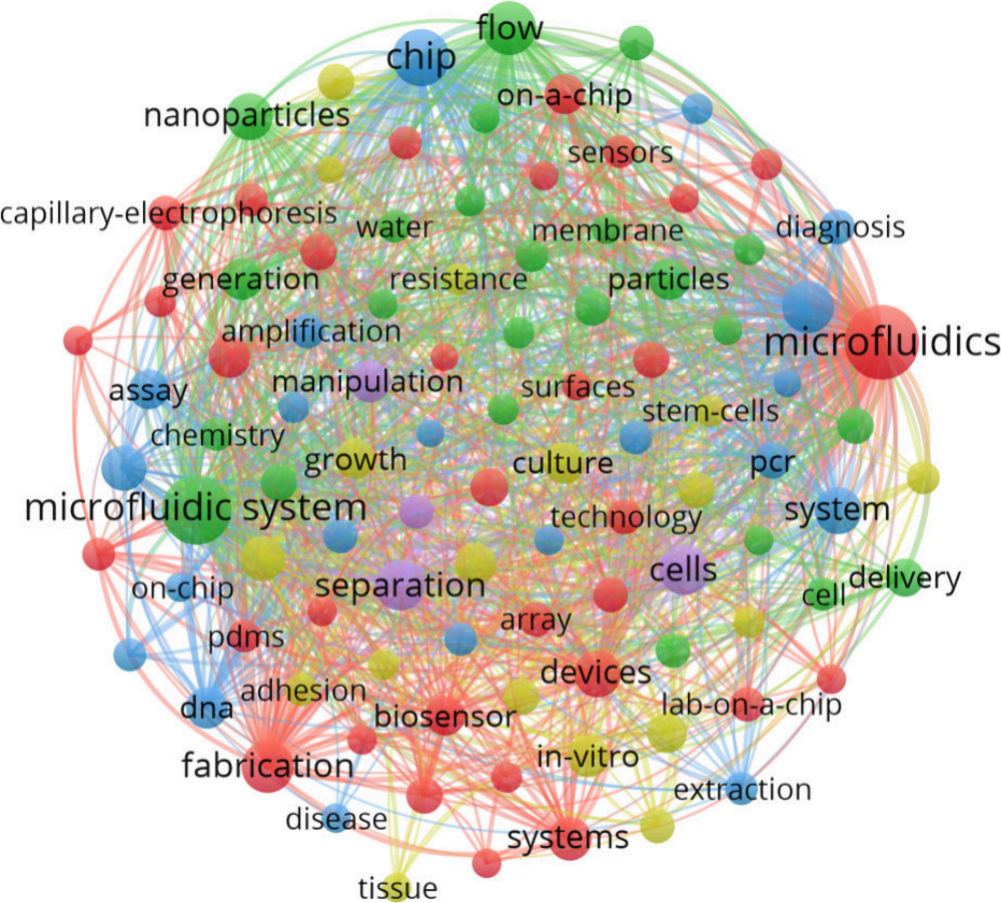
Visualization map of keywords related to the search for keyword occurrence associated with micro and mesofluidic systems.

The analysis of keyword co-occurrence aims to identify emerging and relevant trends. Using VOSviewer, a network map was created to visualize the most frequent keywords. A minimum of 5 occurrences was used to analyze the results, resulting in 1,116 keywords, with Figure 11 presenting the map of the top 100.

As detailed in Table 2, the 20 most prominent keywords in terms of frequency are “microfluidics,” “microfluidic system,” and “chip,” which were the most frequent keywords with 728, 599, and 318 occurrences, respectively, in the inspected documents.

**Table 2:** Classification of the 20 Most Prominent Keywords Identified in the Analyzed Articles

Rank	Keyword	Occurrences	TLS^a^	Rank	Keyword	Occurrences	TLS^a^
1	Microfluidics	728	1751	11	System	177	496
2	Microfluidic system	599	1315	12	Microfluidic	168	357
3	Chip	318	914	13	Platform	161	461
4	Flow	262	725	14	Systems	140	395
5	Device	253	775	15	Manipulation	128	397
6	Fabrication	251	664	16	Culture	127	382
7	Cells	233	641	17	In-vitro	125	280
8	Separation	216	663	18	Generation	123	328
9	Devices	182	514	19	Design	122	351
10	Nanoparticles	180	471	20	DNA	121	371

a TLS: total link strength.

The analysis of the articles in Table 3 shows commonly used processes in micro and mesofluidic systems and their applications. In cluster 1, the review article “From 3D Cell Culture to Organs-on-Chips” addresses techniques focused on cell biology. The study shows the advances in 3D cell cultures and the creation of organs-on-chips, which are microfluidic devices that allow the study of human physiology in a specific organ context. This technology aims to replicate the microstructure, dynamic mechanical properties, and biochemical functionalities of living organs, providing a better understanding of organ function and developing specialized in vitro disease models. Additionally, the potential of organs-on-chips to replace animals in drug development and toxicity testing is discussed.^49^ In the article “Simultaneous Solvent Screening and Reaction Optimization in Microliter Slugs,” the techniques are focused on optimizing chemical reactions. The study addresses the development of an automated system for solvent screening and reaction optimization in microliters using a continuous droplet flow system. The segmented flow analysis suggests a correlation between yield and the hydrogen bonding basicity of the solvent. The study demonstrates the application of an optimized design of experiments strategy for the alkylation of 1,2-diamino cyclohexane in 16 μL droplets. It also highlights the system’s scalability and the limitations for developing and scaling up chemical reactions in the pharmaceutical industry.^56^

**Table 3:** Clusters Based on Bibliometric Analysis

Cluster	Main Keywords	Frequency	Representative articles
1	microfluidic system - conf 83.8% flow - conf 66.7% cells - conf 64.8%	123	(49), (56)
2	chip - conf 95.8% device - conf 60.3% fabrication - conf 52.8%	127	(57), (58)

Cluster 2 focuses on catalytic microchemistry and bioanalysis. The article “Practical approach for macroporous structure embedded microfluidic system and the catalytic microchemical application” presents a practical and low-cost method for generating three-dimensional macroporous structures embedded in microfluidic systems. It describes the fabrication of robust fluorinated microstructures with ordered macroporosity in microfluidic channels, using top-down and bottom-up fabrication techniques without special equipment. In addition, it explores the implementation of lead (Pd) nanoparticles in porous structures for catalytic microchemistry applications.^57^ The article “Cell Lysis and Protein Extraction in a Microfluidic Device with Detection by a Fluorogenic Enzyme Assay” deals with cell lysis and protein extraction in a microfluidic device with a fluorogenic enzyme assay. It describes the successful integration of lysis and fractionation steps on a chip, demonstrating the detection and quantification of β-galactosidase using a fluorogenic enzyme assay. In addition, it discusses the importance of accurate modeling of mass transport and enzymatic reactions in developing these microfluidic devices.^58^

**Figure 12:**
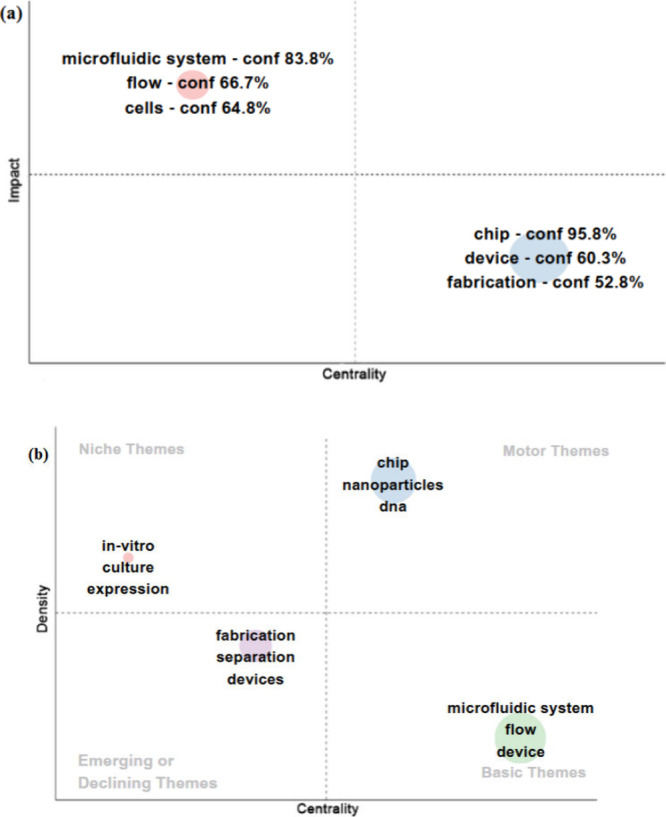
Bibliometric analysis of research on micro and mesofluidic systems. (a) Maps of the main clusters correlating the impact and centrality of the theme. (b) Thematic map correlating the emerging, niche, driving, and basic themes.

Figure 12 was generated from data obtained by Bibliometrix, which presents the analysis of clusters based on the occurrence of keywords, allowing the visualization of the relationship between the most frequent themes in the research, thus showing its relevance and impact. Figure 12–(a) illustrates two main groups of keywords, which are distributed according to impact and centrality. In the upper left quadrant are “microfluidic system” (83.8%), “flow” (66.7%), and “cells” (64.8%), which have a high impact and a lower centrality, which indicates that they are very relevant themes, but are less interconnected with other areas of research. In the lower right quadrant, we have “chip” (95.8%), “device” (60.3%), and “fabrication” (52.8%), presenting a high centrality, which suggests that they play an essential role in the structure of the field of study. Figure 12–(b) illustrates clusters divided into four groups according to density and centrality. Motor Themes include highly central and dense keywords, which implies consolidated topics of great relevance in the area. Niche Themes show words with a high density but a lower centrality, which can indicate specialized issues and growth in specific fields. The Emerging or Declining Themes feature words suggesting areas that may be undergoing change or losing relevance. The Basic Themes indicate that the concepts are essential and widely used in research, thus working as a way to develop new studies.

## 4 Patents Related to Micro and Mesofluidic Systems

In addition to the academic sector, the industrial sector presents significant potential characteristics in using micro and mesofluidic systems. This trend is shown through the analysis of the patent database, which provides an overview of the patents on the topic under study.

**Figure 13:**
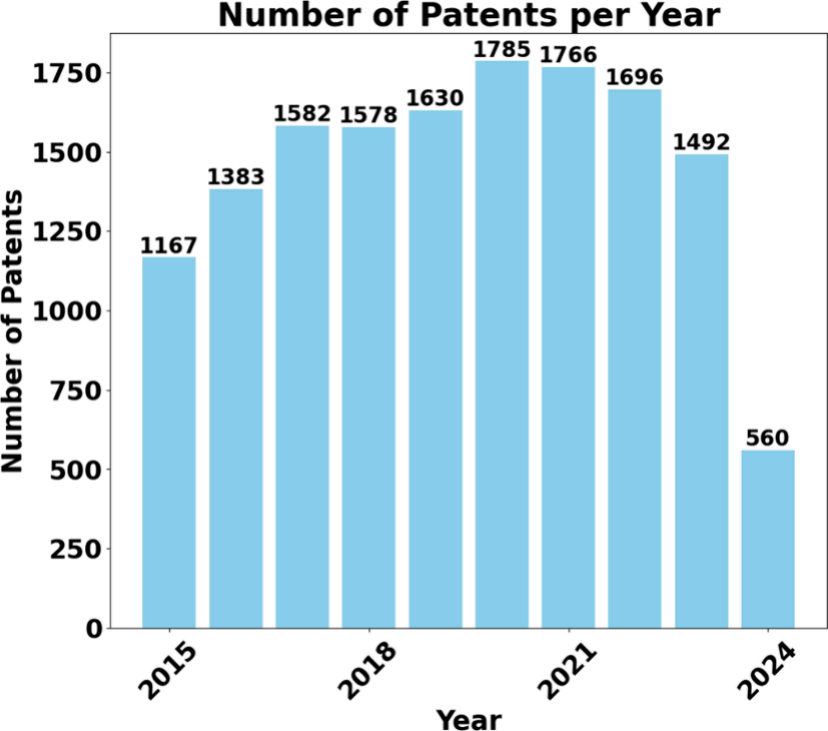
Chronological evolution of patent records over the past 10 years.

A review of the patent database identified 28,212 patents in the area searched. Figure 13 shows the chronological distribution of patents over the past 10 years. Noticeably, every year analyzed, the number of patents exceeds the value of 1,000, except for 2024, as this research is carried out in the first half of the year. It is also worth highlighting the year 2020 for having the highest number of patents registered, totaling 1,785 registrations.

**Figure 14:**
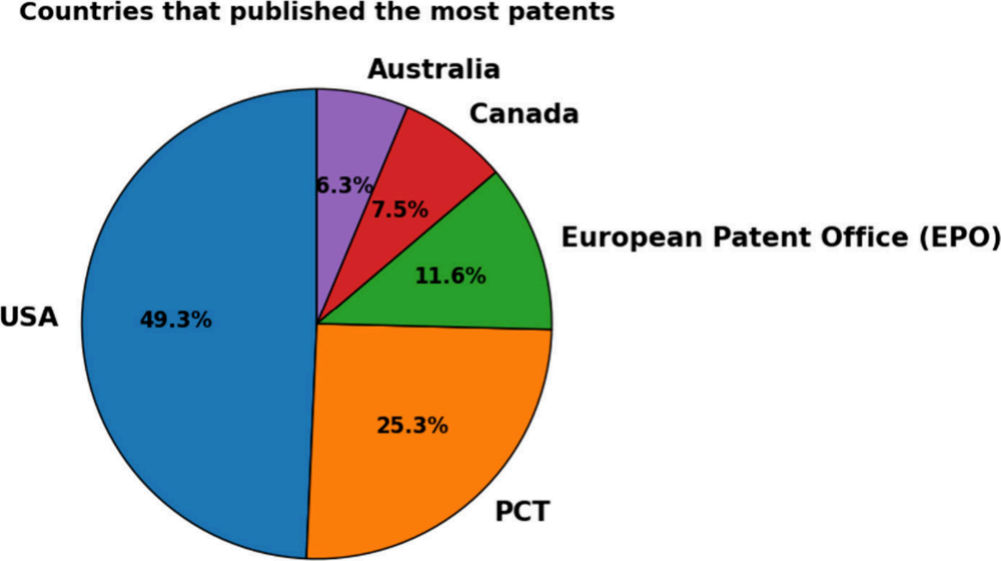
Countries with the most published patents.

Figure 14 shows the distribution of patents by country. The US has the highest number of patents filed, with 12. 995 (49.3%) of the patents, attributing to the USA’s prominence as the leading innovative country in the micro and mesofluidic systems field, in addition to indicating the advance in strategic areas and focus on patents to protect innovations.

Next, the Cooperative Patent System (PCT) presents the registration of 6,668 (25.3%) patents, which reinforces the multiple registrations in partner countries, signaling that several inventors and companies choose to protect their innovations on a large international scale, offering more flexibility for inventors and companies that wish to protect their inventions at a global level.

Another organization responsible for granting patents is the European Patent Office (EPO), with 3,057 (11.6%) patents specifically protecting patents in European countries through a single application. This result indicates that Europe is also an important center for research and innovation in micro- and mesofluidics, although on a smaller scale than the USA.

It is also worth highlighting in Figure 14 countries such as Canada, with 1,976 (7.5%) patents, and Australia, with the registration of 1,660 (6.3%) patents. These results show that both occupy prominent positions, evidencing a focus on research developed in the area.

**Figure 15:**
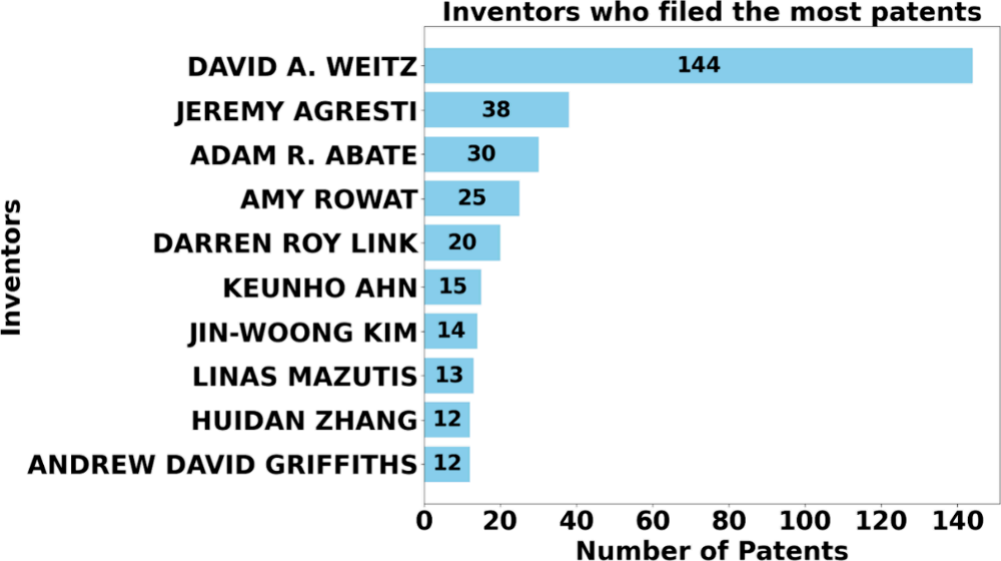
Inventors with the most registered patents.

Figure 15 shows the top ten micro and mesofluidic inventors, classifying them as David A. Weitz, with 144 patents, leading to a significant difference in the other inventors and a possible more substantial impact on the area. Indicating a solid influence and tremendous contributions to advances in micro and mesofluidics, it is also important to highlight that through the high number of patents, Weitz possibly guarantees the exclusivity of its technologies in industrial and scientific applications.

Second, with 38 patents, Jeremy Agresti is far behind Weitz but still contributes substantially, even on a smaller scale, in specific projects involving micro- and mesofluidics. Next, Adam R. Abate appears with 30 patent registrations, suggesting important activities in research and development in this technological sector.

Amy Rowat is also a prominent inventor, with 25 patents in this segment. They were followed by Darren Roy Link, who registered 20 patents, indicating an active research trajectory. They are following other inventors with registrations between 15 and 12 patents, although with a smaller number of patents, their presence suggests collaboration in specific projects aimed at this niche.

**Figure 16:**
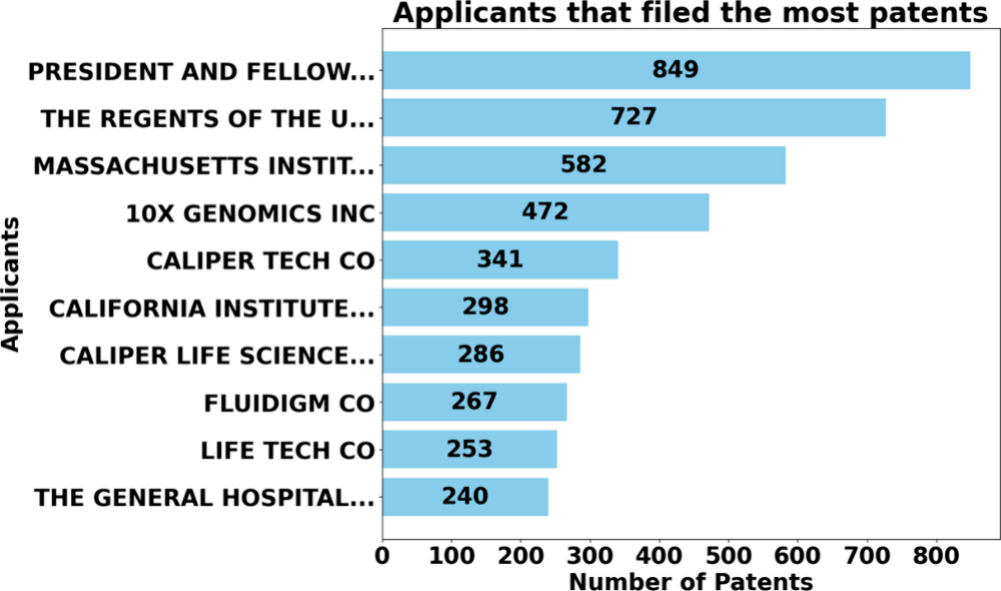
Classification of the leading applicants.

Figure 16 presents the principal patent applicants in detail. The President and Fellows of Harvard College lead with the most significant patent registration, adding up to 849 registrations. This reflects Harvard’s leadership as one of the leading academic institutions in innovation.

Next, the University of California appears to occupy the second position, with 727 patents. This amount portrays the significant collaboration of the California university system, which brings several institutions, such as UC Berkeley and UC San Diego, recognized for cutting-edge research in biotechnology and microfluidics. The Massachusetts Institute of Technology (MIT) is in third place in the ranking, with 582 patents filed, reinforcing its position as an academic and innovation leader.

In fourth position appears a private company standing out among academic institutions, with a total of 472 patents, 10X Genomics is recognized for its sequencing and genetic analysis technologies, and its presence on this list suggests that the company invests heavily in microfluidic systems. Another company that stands out in the ranking of applicants, with a registration of 341 patents, is Caliper Technology Corporation. It has a history of development in microfluidic technologies, including biological analysis devices.

In summary, Figure 16 shows both academic institutions and companies as patent applicants. Where the top three positions are held by educational institutions (Harvard, University of California, and MIT). From the fourth position, companies such as 10X Genomics Inc., Caliper Technology Corp., and Fluidigm Corp., among others, stand out, underscoring the interest and investment of the public and private sectors in protecting innovations related to micro and mesofluidic technology.

## 5 Theoretical Foundations

### 5.1 Physical and Chemical Principles

Microfluidic techniques are broadly classified into two groups: active and passive methods. Active methods utilize an additional external force field, such as acoustic, magnetic, or electric, to achieve particle focusing and separation.^59^ In contrast, passive methods, known as inertial microfluidics, do not require external forces; instead, separation is accomplished through hydrodynamic forces induced by inertial effects and secondary flows intrinsic to microchannel geometries.^60^

The event of focusing and separating suspended microentities based on their geometric and physical properties using microchannels depends on several factors. These factors are the flow condition within the channel that can be characterized in terms of dimensionless Reynolds number, given by eq 1, and the particle size factor expressed as the ratio of the particle diameter to the characteristic length scale of the channel (*a*_*p*_/*L*_*c*_).^59^
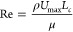
1Here *U*_max_ is the maximum velocity within the channel, and one is the dynamic viscosity of the carrier fluid.^59^

However, there are two definitions for the Reynolds number in microchannels: the mass Reynolds number, shown in eq 2, and the Reynolds number of particles, shown in eq 3.^60^
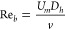
2
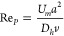
3where *U*_*m*_ is the maximum velocity of the channel, *D*_*h*_ is the hydraulic diameter, a is the diameter of the particle, and v is the kinematic viscosity.^60^

#### 5.1.1 Fluid Dynamics at the Microscale

The dynamics research study identified that the hybrid design better separates neutral and nonfloating particles by providing particle-free samples in more than two output branches. Still, the particle can experience a lagging effect as it approaches the wall, creating an asymmetric flow field around the particles. This results in a repulsive force that pushes the particle away from the wall. The balance of these two forces results in a stable equilibrium position near the channel walls. In a rectangular microchannel, the resulting lift force acting on a spherical particle with neutral fluctuation can be expressed as a function of five dimensionless quantities, as given in eq 4.^59^
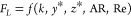
4

The additional contribution of particle density is considered as ρ*, which is the ratio of particle density (*ρ*_*p*_) to fluid density (ρ).^59^

Other researchers add to the studies of microfluidic dynamics and point out that particles in finite and confined Reynolds flows exhibit patterns of lateral focusing and longitudinal ordering. Research also points out that the particle–particle interactions’ magnitude and the resulting pairs’ stability are significantly affected by the transverse geometric arrangement of the pair of particles by the fact that they attract each other, repel or interact minimally. Focused particles attracted, repelled, or minimally interacted with each other based on their geometric arrangement and the distance between particles.^61^ In addition, the particles can self-assemble into longitudinal pairs in a rowed train through flow-mediated interactions.^62^

An interesting study related to fluid dynamics at the microscale is the directional movement of droplets in conical structures, such as tubes and fibers, where geometric confinement directly influences the mobility of droplets. The direction of this controlled movement is due to the inclination of the angle of the structure and the interaction of capillary forces and surface tension, which is an essential phenomenon since there is a need for precision for manipulating small volumes of liquid.^63^ In particular, these structures used in microfluidic devices are efficient in dividing droplets, thus allowing the production of subgroups with precise and controlled volume. Therefore, as a benefit of this fine manipulation of droplets, there is the expansion to the development of advanced system design, such as in the analysis of chemical and biological reactions.^64^

#### 5.1.2 Surface Tension

Studies on surface tension address the dynamics of droplet breakup during the necking stage, which is controlled by the viscous stresses of both phases and the surface tension. The minimum width of the droplet neck decreases linearly with time, and the coefficient of the linear function depends on λ. A critical capillary number of 0.03 is observed in low-viscosity droplets, which delineates the variation in the size of the primary satellite droplet with the capillary number in the constant area and growth zone.^65^

Taking into account that the yarn rupture phase, similar to the formation of high-viscosity droplets on a macroscopic scale and the rupture of the filament in a microfluidic flux focus device, the variation of de *w*_*m*_/*w*_*c*_ with relative time (*t*–*t*_*P*_) can be scaled by a linear relationship, as shown in eq 5, where the linear coefficient k is not constant.^65^
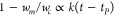
5

#### 5.1.3 Capillarity

In general terms, the phenomenon of capillary action or absorption is the ability of a liquid to flow into narrow spaces without the assistance of external forces through spontaneous movement based on cohesive forces within the liquid and its surroundings. Another term closely related to capillarity is wetting or nonwetting on solid surfaces.^66,67^

Capillary length correlates with two first factors: surface tension force and gravitational force. Capillary length plays an essential role in the shape of a liquid marble concerning stability, as particles at the liquid–air interface introduce a meniscus to the droplet’s surface. In this context, the capillary interaction between the particles can affect the balance of forces on the surface of the liquid and modify the surface tension of a liquid marble. The modified surface tension of a liquid marble is popularly termed the ’effective surface tension’ of the liquid marble, formulated by eq 6.^68^
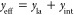
6where *y*_eff_ is the adequate surface tension of the liquid marble, *y*_la_ is the surface tension at the liquid–air interface and *y*_int_ is the contribution to the adequate surface tension due to capillary and electrostatic interactions.^68^

T particles’ size and properties also affect the droplet’s surface energy.^50^ Another necessary consequence of capillarity in the contact line is the dynamic contact angle induced by the movement of the droplet.^69^

Surface tension acts on several fronts, including the Jamin effect. This phenomenon occurs when tiny droplets or bubbles need to move in confined spaces, such as capillaries or narrow tubes, which has been improved by adding solutions such as sodium dodecyl sulfate (SDS) to accelerate the capillarization process.^70^

#### 5.1.4 Scale Effects

Scale effects in microfluidics refer to the changes in fluid behavior when the dimensions of fluidic channels are reduced to the micrometer scale. These effects arise from the different physical forces that dominate at this scale. These include the Reynolds number (Re), which addresses inertial effects; the Péclet number (Pe), related to convective and diffusive transport; the capillary number (Ca), expressing the importance of interfacial tension; the Deborah, Weissenberg, and elasticity numbers (De, Wi, and El), which describe elastic effects due to deformable microstructural elements such as polymers; the Grashof and Rayleigh numbers (Gr and Ra), describing density-driven flows; and the Knudsen number, which highlights the importance of noncontinuum molecular effects.^3^ It is worth noting that elastopillary number and capillary length, both complementary and relevant parameters for microfluidic systems, explain the relationships between elastic forces and surface tension.^63−65^ The dimensionless numbers are shown in Table 4.

**Table 4:** Dimensionless Numbers in Microfluidics

Acronym	Dimensionless Numbers	Mais Description	Mathematical Formula	Equation Number
Re	Reynolds	inertial/viscous	*ρU*_0_*L*_0_/η	Equation 8
Pe	Péclet	convection/diffusion	*U*_0_*L*_0_/*D*	Equation 9
Ca	Capillary	viscous/interfacial	*ηU*_0_/γ	Equation 10
Wi	Weissenberg	polymer relaxation time/shear rate time	*τ*_*P*_/γ	Equation 11
De	Deborah	polymer relaxation time/flow time	*τ*_*P*_/*τ*_flow_	Equation 12
El	Elasticity	elastic effects/inertial effects	*τ*_*P*_*η*/*ρh*^2^	Equation 13
Gr	Grashof	Re for buoyant flow	*ρU*_*b*_*L*_0_/η	Equation 14
Ra	Rayleigh	Pe for buoyant flow	*U*_*b*_*L*_0_/*D*	Equation 15
Kn	Knudsen	slip length/macroscopic length	β/*L*_0_	Equation 16

Where the Reynolds number (Re), relates the inertial forces to the viscous forces; the Péclet number (Pe), associates convection with diffusion; the capillary number (Ca), links viscous forces with surface tension; the numbers of Deborah (De), Weissenberg (Wi) and elasticity (El), express elastic effects; the Grashof (Gr) and Rayleigh (Ra) numbers, connect transport mechanisms in buoyancy-driven flows; and the Knudsen number (Kn), correlates microscopic with macroscopic length scales.^3^

Table 4 is an adapted version of TABLE 1. Dimensionless numbers used in this review.^3^ A header highlighted the column of acronyms, dimensionless numbers, main descriptions, mathematical formulas, and equation numbers.

To complement, surface tension strength plays a vital role when the length scale of a system is small (i.e., in the micro/nanometer range). In the case of compliant structures and soft substrates, the competition between interfacial energy and elastic strain energy in volume gives rise to many exciting phenomena where capillary length can influence the stability of droplets and interfaces in microfluidic systems.^71^ However, elastocapillarity refers to the study of the effects of the deformation of flexible structures or soft substrates under the action of capillary forces. The elastopillary interaction between liquid and solid can give rise to adhesion in microstructures, droplet envelopment by flexible structures, fiber bending, and ridge formation in the contact line of a droplet on a soft substrate. Recent research has shown that elastocapillarity aids in manipulating liquid flow through flexible confinements and over soft substrates. With these soft materials and experimental methods, studying the rich physics of elastocapillarity was possible. For example, droplets can interact with each other through a substrate or sense tension in the underlying substrate. In comparison, capillary microfluidics uses passive devices, where fluid flow is self-conducted and accomplished with surface tension force and wettability.^71−73^

These scale effects are critical to developing and optimizing microfluidic devices, enabling chemical analysis, biology, medicine, and engineering applications.

## 6 Technologies and Tools

### 6.1 Microfluidic Devices

Microfluidic devices are used in various fields of science and technology. They allow for the precise manipulation of fluids at micrometer and nanometer scales. Among the main types of microfluidic devices are microfluidic channels. These transport and mix small volumes of fluids and have been utilized in many biological applications, including high-density rapid sequencing, polymerase chain reaction (PCR), and single-molecule detection. They consist of channels with micrometric dimensions sculpted into materials such as polydimethylsiloxane (PDMS), glass, or silicone;^74^ artificial blood vessels are employed in biomedical research to simulate blood circulation and study the interaction between cells and blood flow. This technique involves constructing a microvascular network that mimics cellular activities in organs using traditional or existing fabrication techniques;^75^ separation microchips, this method shows promise for developing an integrated lab-on-a-chip device. Critical applications include capillary electrophoresis and liquid chromatography, aimed at analyzing preconcentration, separation, and detection of various compounds within a single device;^76^ micromixers, unlike separation microchips, micromixers are typical devices used to mix small amounts of fluids using various mixing principles, either actively or passively. Active mixing requires energy sources such as acoustic, electrokinetic, electrowetting, magnetic, or electromagnetic forces, while passive mixing does not require external energy sources;^77^ microreactors, used in chemistry to carry out chemical reactions on a small scale with high efficiency, the miniaturization of the reactor results in a very high surface area-to-volume ratio, ranging from 10,000 to 50,000 m^–1^, making it more efficient compared to traditional chemical reactors;^78^ microsensors, these devices incorporate sensors to detect and measure physical, chemical, or biological properties of fluids. Examples include glucose sensors, biosensors, and pH sensors such as Ion-Selective Field Effect Transistors (ISFETs), which are particularly useful for measuring pH and other ions in small volumes and can be integrated into compact flow cells for continuous measurements;^79^ lab-on-a-chip systems, these integrated systems combine several laboratory functions on a single chip, including sample preparation, reaction, separation, and detection. It was recently discovered that the efficiency of magnetic particle separation in lab-on-a-chip devices increased from 50.2% to 91.7% and decreased from 88.6% to 85.7% within the range of depth factors from 15 to 27 μm and width factors from 30 to 60 μm;^80^ microvalves, these can open or close channels to direct fluid flow in two ways: actively, using mechanical and nonmechanical moving parts as well as external systems, and passively, using mechanical and nonmechanical moving parts. Micropumps, on the other hand, use a variety of interaction phenomena between an electromagnetic field and the working fluid to generate pressure and flow;^81^ microchambers for cell culture, this technique is widely used to cultivate and analyze cells in a controlled environment that allows for the engineering of structurally organized tissues, guiding the growth of cellular spheroids within matrices in microchambers.^82^ Microneedlesave emerged as a unique delivery technology, offering a promising medication administration pathway, especially in complex organs such as various ocular conditions.^83^

### 6.2 Instrumentation and Equipment

The main instruments and equipment for manipulating and analyzing micro and mesofluidics are often employed to characterize experimental samples.

For instance, microscopy is fundamental in scientific research, diagnosis, technological development, and education. Given that microscopy encompasses a wide range of analytical devices, it is essential to highlight the primary ones. Optical microscopy is generally easy to use and allows for the analysis of samples in natural colors, both in air and water, making it applicable across various fields of knowledge. However, compared to Scanning Electron Microscopy (SEM), optical microscopy has limitations regarding magnification and resolution capabilities.^84^ Another widely used technique in microscopy is transmission electron microscopy (TEM), which is employed to observe changes in materials after specific processes, such as catalyst degradation or the wear of filtration membranes.^85^

Spectroscopy offers a wide range of equipment models. One of them is vibrational spectroscopy, a nondestructive technique that measures the vibrational energy within a compound, functioning as a unique fingerprint to determine the structures of compounds. The main methods include mid-infrared and Raman spectroscopy, both related to the fundamental vibrations of molecules and used to identify the molecular structure, dynamics, and surrounding environment of compounds.^86^

### 6.3 Materials and Fabrication

The fabrication of microfluidic devices involves various materials, with the choice depending on the specific needs of the microfluidic device, including factors such as the nature of the fluids being manipulated, optical transparency requirements, chemical compatibility, flexibility, cost, and ease of fabrication.

The primary substrates used for fabricating microchannels include glass slides, known for their high chemical resistance, excellent optical transparency, and good compatibility with high pressures and temperatures; silicon wafers, which offer high fabrication precision (using semiconductor microfabrication techniques) and good thermal conductivity.^87^ Polydimethylsiloxane (PDMS), this material is a common choice for fabricating microfluidic devices due to its transparency, flexibility, biocompatibility, low cost, and ease of molding;^88^ poly(methyl methacrylate) (PMMA), An excellent candidate for microengineering to control the microenvironment of stem cells, with advantages such as good optical transparency, ease of molding, and biocompatibility;^89^ polyethylene terephthalate (PET), This material has potential applications in microfluidics and organ-on-chip systems, where cells grown on porous substrates are used to simulate biological barriers and transport processes;^90^ microfluidic polyurethane, this material is accessible and has significant potential as an alternative to PDMS or thermoplastics for fabricating microfluidic devices;^91^ poly(lactic acid) (PLA), this material is notable for being biodegradable, in addition to having numerous scientific and practical applications.^92^

#### 6.3.1 Lithography

Lithography is a fabrication technique that uses light to transfer a pattern from a mask onto a light-sensitive surface, typically a substrate coated with a thin film of photoresist material. Several lithography techniques, including optical lithography (photolithography), use ultraviolet (UV) light to expose a photoresist layer on a substrate. The light passes through a mask that defines the desired pattern;^93^ X-ray lithography, this technique combines the ability to penetrate thick resins and produce high aspect ratio patterns, achieving sidewalls with optical quality roughness and offering higher resolution than optical lithography due to the shorter wavelength of X-rays;^94^ electron beam lithography, this method has several advantages, such as combining high-performance patterning capabilities over large areas, feature sizes down to the submicrometer range, and the ability to write directly onto the photoresist without the need for a mask, providing high resolution;^95^ nanoimprint lithography, this technique offers several technical advantages, including lower imprint pressure and the elimination of thermal cycles, making it crucial for the fabrication of multifunctional devices and promoting the convergence of various technological disciplines;^96^ interference lithography, used to create large-scale, high-resolution periodic structures, with applications in photonic crystals, metamaterials, plasmonic nanostructures, wire grid polarizers, and more;^97^ projection lithography, over the years, projection lithography has been scaled by increasing the numerical aperture (NA) of the projection lens, reducing the wavelength (ƛ), and decreasing *k*_1_, which can be influenced by various optical techniques such as phase-shifting masks and off-axis illumination. Moreover, optical projection lithography will remain essential for fabricating advanced chips.^98^

Although lithography has remained the state-of-the-art method for producing microfluidic (MF) chips for over 30 years, it has certain drawbacks, including the high costs of using expensive silicon wafers, sophisticated machinery, stringent cleanroom requirements, and lower production volumes.^99^

#### 6.3.2 3D Printing

3D printing of microfluidic systems represents a significant advancement in the fabrication of biocompatible and complex devices, with diverse applications in biology, materials engineering, and tissue engineering. One study highlighted the use of polymers such as poly(lactic acid) (PLA) and acrylonitrile butadiene styrene (ABS), printable via Fused Deposition Modeling (FDM). These materials proved effective for cell adhesion and proliferation after surface modifications, such as alkaline hydrolysis and thin film deposition.^100^ Another study emphasized microfluidic techniques for creating porous and ordered scaffolds, precisely controlling pore size and distribution, which is essential for applications in tissue engineering and regenerative medicine.^101^

The evolution of this fabrication method has revolutionized microfluidic systems, providing unprecedented design flexibility and the ability to create complex and multifunctional devices. 3D printing enables the production of essential devices for biotechnological applications,^102^ and its advances in drop-based printing highlight the rapid and cost-effective fabrication of precise channels and complex internal structures, which are fundamental for droplet manipulation in biology and analytical chemistry.^103^ Furthermore, challenges overcome by 3D printing, such as creating accurate and flexible three-dimensional architectures, allow for an efficient transition from prototype to large-scale manufacturing of microfluidic devices.^104^

#### 6.3.3 Micromolding

Micromolding was introduced into microfluidics in the 1990s to reduce the cost per chip and expand the range of materials previously limited to silicon and glass. Micromolding enables many microfluidic applications that are impossible with silicon or glass micromachining.^105^ Today, most microfluidic devices are manufactured using micromolding processes. These processes work very well for a limited set of biocompatible materials.^106^

In general, micro molding is highlighted as an efficient and low-cost technique for fabricating complex microscale structures, allowing the integration of optical components into microfluidic devices for various applications, such as micro total analysis systems (μTAS) and lab-on-a-chip (LOC) systems.^107^

The versatility of Fused Deposition Modeling (FDM) in creating complex and multifunctional microfluidic devices. FDM is a 3D printing technique that uses thermoplastic material to build objects layer by layer. One advantage of micro molding is the ability to produce complex parts with high precision. However, there are challenges, such as the need for advanced techniques to create precise molds and the difficulty in maintaining the structural integrity of components during the molding process.^107^

## 7 Recent Advances

### 7.1 New Technologies and Materials

A combination of technological innovations has driven recent advances in micro and mesofluidics. Recent studies have investigated the scaling of thermal micropumps driven by thermal bubbles from the meso to the microscale. These micropumps are emerging technologies that can be directly integrated into micro/mesofluidic channels and do not have moving parts, thus taking advantage of existing mass manufacturing methods.^108^

Other recently reported advances include methods for liquid control and the applications of both open and closed microfluidics in biosensors. It is essential to distinguish between open and closed microfluidics, as both have unique methods of liquid manipulation and various applications in biosensors. Open microfluidics can be highlighted for immunoassays, single-cell analysis, biomolecule analysis, drug screening, and medical diagnostics, emphasizing precision and integration with analytical methods. In contrast, closed microfluidics addresses the fundamentals and techniques of liquid flow control, such as the Reynolds number, Péclet number, capillary number, and Weber number.^2^

Research on the relationship between wettability and microfluidics is currently being explored, as the modulation of wettability can significantly influence microfluidic systems used in various fields, such as signal detection, cell culture, and material synthesis. In general, wettability theory aims to examine natural phenomena such as the superhydrophobicity of plants and the adhesive capability of gecko feet, which are used to inspire materials with specific wettability properties.^109^

Another current possibility is the integration of Artificial Intelligence (AI) and Machine Learning (ML) in these systems. For example, we have the automation of microfluidic systems with AI and ML for diagnosis and monitoring, where a microfluidic system could optimize data analysis in real-time and automatically adjust parameters to increase accuracy and reduce reagent consumption. It would also be possible to detect patterns in large data volumes, facilitating automated diagnostics and monitoring.^110,111^

These systems also increase the use of advanced and sustainable materials, thus enabling high performance and reducing environmental impact. In this context, we have advanced microfluidic systems to capture circulating tumor cells, using biocompatible and recyclable materials such as poly(ε-caprolactone) (PCL) and Fe_3_O_4_ nanoparticles that combine functionality and sustainability in the design of biomedical devices.^112^ Another similar advance is the nanoparticle manipulation systems that use dielectrophoretic forces, reducing energy consumption and being more efficient and sustainable.^113^

Based on these advances, strategies were employed to develop an innovative dialysis configuration, one of which was the fabrication of a microfluidic system containing a three-dimensionally (3D) printed microchannel filter composed of a polycaprolactone/Fe_3_O_4_ nanoparticle composite with a pore diameter of less than 200 μm. In this system, a high-voltage magnetic field is focused on enriching and isolating circulating tumor cells (CTCs) targeted by nanoparticles from a large blood volume. Under optimized conditions, this dialysis system exhibited more than 80% efficiency in isolating CTCs from blood samples.^112^

**Figure 17:**
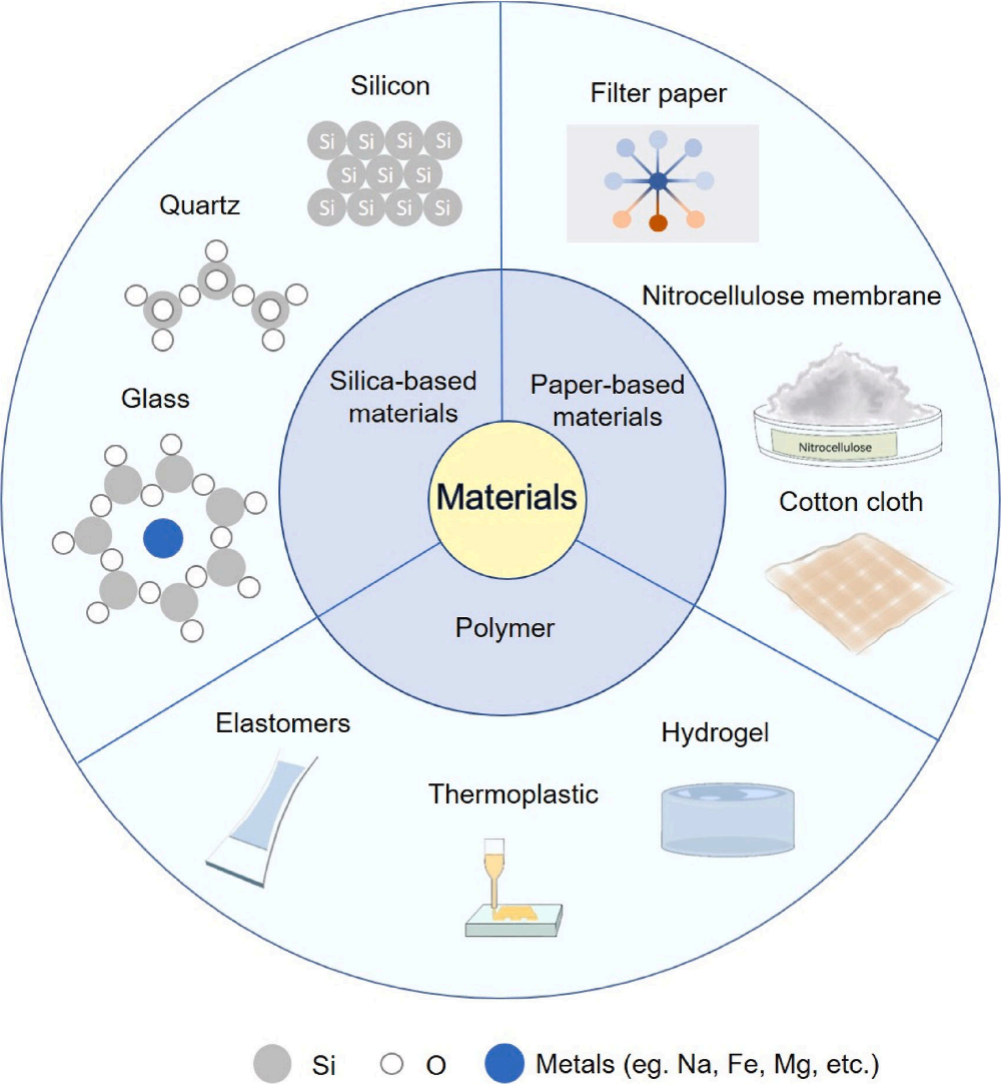
Substrate materials used in microfluidic chips.^2^

In addition to advances in microfluidic dialysis systems, microfluidic chips play a fundamental role in various applications and are manufactured using different substrates. The three most commonly used substrates are silica-based, polymer-based, and paper-based, as shown in Figure 17. Silica-based materials commonly used include monocrystalline silicon, glass, and quartz. Polymers in lab-on-a-chip systems vary and can be classified into thermoplastics, elastomers, and hydrogels. Paper-based microfluidic chips typically use filter paper, nitrocellulose membrane, and cotton fabric as substrate materials.^2^

In microfluidic technology, the limited reaction space facilitates rapid kinetics, efficient reagent utilization, and the synthesis of a wide range of complex organic compounds, inorganic materials, and biomolecules. Digital microfluidics provides a promising platform for microfluidic synthesis, and electrowetting-based electrophoretic manipulation is becoming a common method for controlling microfluidic processes.^114^

In addition, nanogenerators, especially triboelectric ones, are also used to feed these systems autonomously, thus taking advantage of the energy of the environment and human movement. Such technology dispenses with conventional sources and reduces costs.^114^ These examples show that these materials and sources can make micro and mesofluidic systems more efficient and environmentally friendly.

Table 5 highlights the primary materials used in micro and mesofluidic systems, presenting their properties, applications, and corresponding references. Among the materials listed in the table are silica, polymer, and paper-based substrates, each with specific characteristics that influence their applicability. Materials such as monocrystalline silicon, glass, and quartz are widely used due to their precision, thermal stability, and chemical resistance, making them essential for high-precision devices. Polymers, including thermoplastics, elastomers, and hydrogels, offer flexibility, low cost, and biocompatibility, making them ideal for lab-on-a-chip devices and biomedical microfluidic systems. Additionally, the use of Fe_3_O_4_ magnetic nanoparticles and poly(ε-caprolactone) (PCL) is emphasized, as they are fundamental for selective capture systems of circulating tumor cells (CTCs) and tissue engineering.^112,115−117^ Thus, the table presents the main material options and their contributions to the advancement of microfluidic technology.

**Table 5:** Materials Used in Micro and Mesofluidic Systems

Material	Properties	Applications	Reference
Poly(ε-caprolactone) (PCL)	Biocompatible, biodegradable, can be used in 3D printing	Microchannel structures, tissue engineering	(112)
Fe_3_O_4_ Nanoparticles	Magnetic properties, used for selective cell capture	Circulating tumor cell (CTC) capture, magnetic separation	(112)
Monocrystalline Silicon	High precision, thermal and mechanical stability	High-precision microfluidic devices	(115)
Glass	Optical transparency, chemical resistance	Optical and chemical analysis systems	(115)
Quartz	High mechanical strength, thermal stability	Microchips for biomedical applications	(115)
Thermoplastics	Lightweight, flexible, low manufacturing cost	Lab-on-a-chip devices, flexible microchannels	(116)
Elastomers	High elasticity, chemical resistance	Micropumps, flexible valves	(116)
Hydrogel	Biocompatible, can retain moisture	Controlled drug release systems	(116)
Filter Paper	Low cost, biodegradable, porous	Disposable microchips for rapid analysis	(117)
Nitrocellulose Membrane	High retention capacity, applicable in biosensing	Biosensors for clinical and environmental testing	(117)
Cotton Fabric	Flexible, durable, used in portable devices	Filtration and separation in portable devices	(117)

### 7.2 Innovations in Fluid Manipulation: New Methods for Controlling and Manipulating Fluids at Micro and Mesoscales

One of the investigations conducted by researchers today on microfluidics is that most microfluidic flows are triggered by pressure. Consequently, the liquid flow can be laminar turbulent, which may be laminar and turbulent, depending on the force applied. For this, it is necessary to use eq 7, which determines whether the flow is turbulent or laminar by applying the Reynolds number.^4^
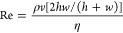
7

*Re* is the dimensionless Reynolds number, ρ is the density, η is the dynamic viscosity of water, *w* is the velocity of linear flow, *h* is the height of the channel, and *w* is the width of the channel.

Another expansive and prominent line of research in the field of microfluidics is the study of microplastic pollution, which is increasing at an alarming rate. Studies focused on wastewater analysis have detected polystyrene with a diameter of 15 μm, PET fibers, and nylon-6 fibers with lengths of 200 μm. An acoustic focusing device was incorporated to collect microplastics by designing a Pyrex glass microfluidic chip with channels 707 μm wide and 505 μm deep.^118,119^

## 8 Main Applications

Microfluidic systems have a wide range of applications across various fields of science and engineering, as demonstrated by the studies analyzed. Biology and biomedicine are used for rapid diagnostics, cell culture, angiogenesis studies, vessel growth, and microorganism manipulation for chemotaxis studies.^120−122^ In chemistry and biochemistry, microfluidics is employed in chemical and biochemical analyses, allowing for reduced use of samples and reagents while providing rapid and precise detections.^115,120,123^ In terms of engineering and manufacturing, these systems are used to fabricate microchannels with adjustable mechanical properties, facilitating fluid-structure interaction studies at the microscale.^120,123^ Additionally, there are innovative applications in biomimetics, where natural surfaces are replicated for droplet control and drag reduction in microchannels.^120^ Finally, in sensors and actuators, microfluidics is essential in developing sensors for monitoring pressure, temperature, and liquid refractive indices.^120,124^

With new advances and developments in various application areas, micropumps have emerged as a promising example of innovation. They are actuated by thermal bubbles and are considered a future micro actuator technology that can be directly integrated into micro/mesofluidic channels. These micropumps have no moving parts and leverage existing mass-production manufacturing approaches. As such, they hold great promise for micro/mesofluidic systems, such as lab-on-a-chip technologies.^108^

**Figure 18:**
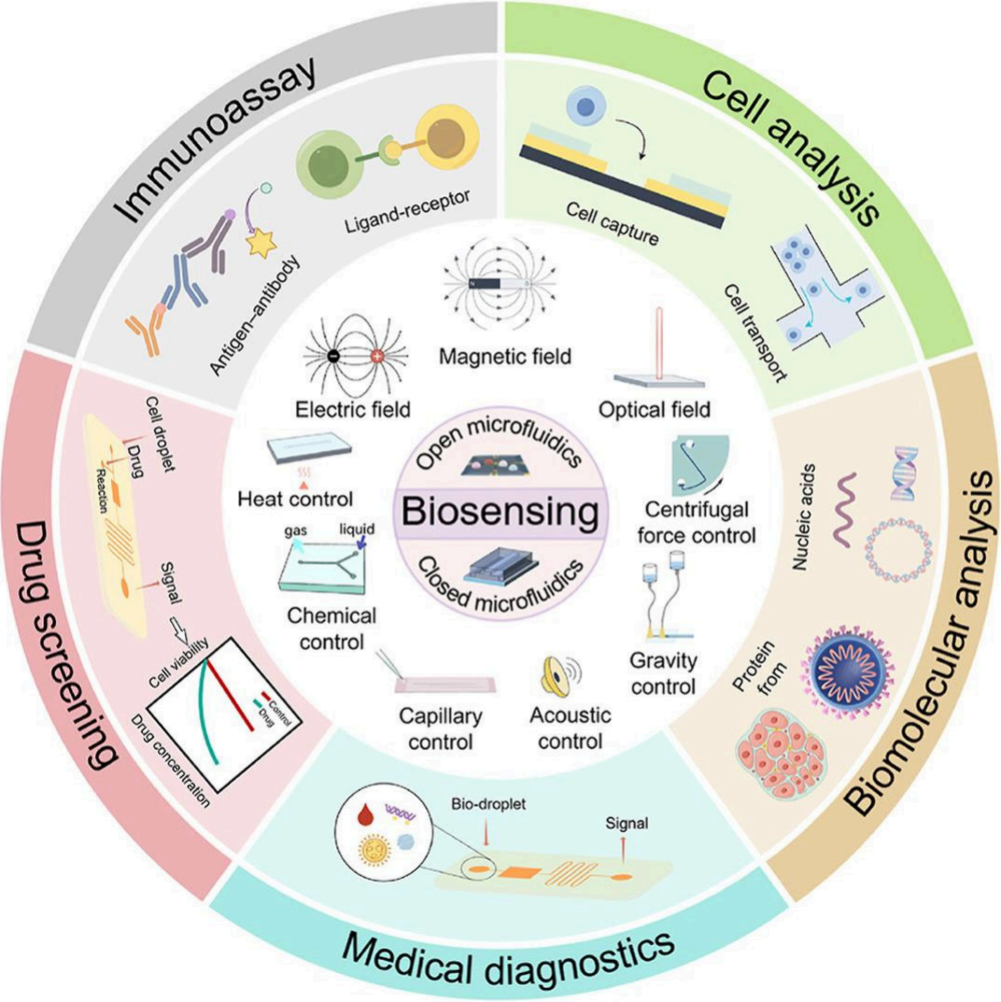
Applications of microfluidics in biosensing, including medical diagnostics, cell analysis, drug screening, and biomolecular detection.^2^

Another example is microfluidics, which has proven to be a powerful tool for biosensing by integrating biological detection processes into a palm-sized chip. Biosensing is vital in many fields, including disease diagnosis, infectious disease prevention, and point-of-care monitoring. It is worth highlighting the ability of microfluidics to combine fundamental biological detection operations, such as sample preparation, mixing, reaction, separation, and detection. This chip, capable of performing multiple functions in conventional biology laboratories, has demonstrated its effectiveness as a biosensing tool and has become one of the most advanced scientific disciplines.^2^Figure 18 presents a schematic diagram of microfluidic applications in biosensing, highlighting different control methods (chemical, thermal, electrical, magnetic, optical, acoustic, and capillary). These techniques enable various applications, such as medical diagnostics, cell analysis, drug screening, biomolecular analysis, and immunoassays. The diagram emphasizes how these processes are integrated into microfluidic systems to enhance the detection and analysis of biomolecules, cells, and therapeutic compounds.

In this context, microfluidic technology is widely used to perform biochemical synthesis methods. It utilizes small-scale microfluidic channels on a microfluidic chip to conduct chemical reactions through microfluid-to-microfluid contact and fusion. This technology offers several advantages. First, it enables precise control over reaction conditions such as temperature, time, and concentration, resulting in high-purity products with high yield.^114^ The droplet-based microfluidic system was used to form each spheroid from four different subtypes of breast cancer cells. This discovery may provide valuable insights into predicting the aggressiveness of unknown tumor cells.^111^ Considering all these as significant advancements.

Table 6 presents the main technological developments discussed in Chapter 8, highlighting the application areas, significant technical advances, and corresponding references. Among these developments, digital microfluidics is used in biomedical analyses and portable diagnostics, enabling precise volume control and reduced reagent consumption. Thermal bubble-driven micropumps represent an innovation in fluid transport within microfluidic systems, allowing high efficiency without moving parts. Meanwhile, microfluidics-based biosensors are essential for biomarker monitoring in disease diagnostics, providing rapid and highly sensitive biomolecule detection. Additionally, microfluidic systems for tumor cell culture create a controlled environment for analyzing cell aggressiveness and testing pharmaceuticals, while microfluidic biochemical synthesis enhances the purity and yield of reactions on a small scale. Finally, nanoparticle manipulation using dielectrophoretic forces enables precise separation with lower energy consumption in miniaturized devices.^108,113,114,124−126^ Thus, the table summarizes the main advances in microfluidics and their applications, highlighting the impact of these technologies on the development of innovative devices.

**Table 6:** Key Technological Developments

Technological Development	Application Area	Technical Advances	Reference
Thermal Bubble-Driven Micropumps	Fluid transport without moving parts in microfluidic devices	High efficiency and integration into miniaturized systems	(108)
Nanoparticle Manipulation with Dielectrophoretic Forces	Separation and precise control of particles in miniaturized devices	Reduced energy consumption and increased precision in particle manipulation	(113)
Microfluidics for Biochemical Synthesis	Optimized chemical reactions on a small scale	Improved purity and reaction yield	(114)
Microfluidics-Based Biosensors	Biomarker monitoring for disease diagnosis	Rapid and sensitive biomolecule detection	(116)
Microfluidic Systems for Tumor Cell Culture	Prediction of tumor cell aggressiveness and drug testing	Controlled environment for cell growth analysis	(127)
Digital Microfluidics	Chemical, biomedical analysis, and portable diagnostics	Precise volume control and lower reagent consumption	(126)

## 9 Main Challenges and Limitations

The technology of 3D cell culture and stem cell biology, particularly organoids, faces several challenges in combining biology and microengineering to become a primary tool in drug research. Current microfluidic systems designed for organoid formation cannot accommodate organoids larger than 400 μm in diameter, making them incompatible with various organ models. Additionally, organoids are significantly lost during the transfer process from cell culture plates to microfluidic devices. This highlights a growing challenge in contemporary medicine related to the increasing number of diseases resistant to treatments and a decline in the development of new drugs.^128^

Another aspect of medical research involves microfluidic chips, which demonstrate superiority over conventional methods for DNA methylation analysis. DNA methylation involves adding a methyl group to the fifth carbon of cytosine within a CpG dinucleotide, generating 5-methylcytosine, an important epigenetic marker. Microfluidic chips allow for the miniaturization and automation of various biological analyses, including precisely quantifying DNA methylation.^125^ One of the most promising techniques for methylation analysis is digital PCR (dPCR), which offers sensitivity and precision in quantifying target molecules, making it useful for detecting rare methylation in normal tissues. However, integrating dPCR into microfluidic chips still faces challenges, such as the miniaturization of reaction components and the generation of many partitions homogeneously.^127^

Microfluidic biochips—a specific type of microfluidic chip designed for biological analyses—also present challenges in integrating all the necessary processes for biomedical analyses and manipulating small volumes of liquids. Concerns regarding these devices’ security and privacy include hacking vulnerabilities, hardware Trojan attacks, design modification, functionality alteration, overproduction by suppliers, counterfeiting, and reverse engineering.^129^

Many devices are made of PDMS due to their rapid curing at room temperature, transparency that allows direct visualization of fluids, and inertness to various chemical and biological reagents. However, PDMS has limitations in its applications due to solvent swelling, absorption of hydrophobic molecules, lack of mechanical rigidity, and low manufacturing yield, making large-scale production challenging.^116,117^

Microfluidic technologies use various physical phenomena to manipulate cells and flows, such as magnetic traps, sound waves, micropatterns, and wettability. This is a crucial property in microfluidics as it determines the intermolecular interactions between liquid and solid. Despite advances in preparing superhydrophobic materials, large-scale application faces difficulties, as such surfaces are delicate and susceptible to mechanical damage and chemical attacks, leading to a loss of properties or even complete failure.^109,117^

Some challenges and limitations of microfluidic chemical phenomena include discrepancies between theoretical models and experimental results. Theoretical models often assume homogeneous materials, which do not reflect experimental reality. The roughness and treatment of microfluidic channel surfaces can influence fluid behavior, and there is vulnerability to contamination and evaporation.^109^

Regarding commercialization barriers, inventors and researchers face the challenge of saturated patents and limited space for new intellectual properties. Investment opportunities are restricted and depend on successful investments at various stages, resulting in a competitive market.^117^

## 10 Future Perspectives

Due to the vast and promising ways of fluid manipulation, a wide range of opportunities and applications for micro and mesofluidic systems are emerging. The key areas include biomedicine and health, through rapid diagnostics and testing for various diseases, as well as organ-on-a-chip devices with functions similar to human organs and single-cell analysis; chemistry and chemical engineering, for conducting chemical reactions with control and efficiency, reducing waste production, and facilitating the production of nanoparticles with controlled size and shape; biotechnology, through high-throughput screening and 3D tissue culture; environmental analysis; for monitoring water and air quality, as well as field sample analysis; electronics and advanced materials, creating compact, multifunctional electronic devices and developing new materials with improved properties; food industry, for quality control and the development of flavors and aromas; nanotechnology, through precise manipulation of nanoparticles and nano biosensors.

The lab-on-a-chip concept offers a range of promising applications across various fields, including clinical diagnostics, food and environmental analysis, and drug discovery and delivery studies.^7^ There is also the case of organ-on-a-chip systems with organoids, where the synergy between organoids and microfluidics can generate more complex and physiologically relevant models. This reduces the reliance on animal models and the costs associated with drug development while enhancing the predictability and safety of development stages.^128^

Another promising segment is digital microfluidics, which holds potential applications in medical diagnostics and analytical chemistry. It is highlighted as an innovative platform that allows individual control of liquid droplets in an open matrix of microelectrodes. This technology benefits the parallel processing of fluid samples, making it ideal for synthesizing and characterizing nanoparticles on a single chip.^126^

Future technologies that could revolutionize the field include fully integrated and self-sufficient systems capable of performing analysis without human intervention after sample insertion. An example is a self-sufficient microfluidic system based on “lab-on-a-foil” for automated nucleic acid analysis using recombinase polymerase isothermal amplification.^130^

Micro and mesofluidic technologies, which involve the precise control of small volumes of fluids, have the potential to revolutionize various sectors, generating significant socioeconomic impacts: healthcare, through Point-of-Care (POC) diagnostic devices that provide rapid and accurate responses, essential for real-time medical diagnostics;^131^ food safety, addressing applications in quality monitoring and the detection of pathogens, allergens, toxins, heavy metals, pesticide residues, and other chemical and physical contaminants in food;^132^ education and research, an example is mixed reality technology, which allows students to visualize invisible microscopic processes, such as chemical reactions, through 3D models and animations, integrating these abstract concepts with practical experiments;^133^ economic development and job creation, for example, microfluidics in biofuel development proposes the miniaturization of experiments with very low solvent, energy, and time consumption, resulting in more precise outcomes.^134^

## 11 Conclusions

Research on micro and mesofluidic systems has demonstrated continuous growth and significant impact in academia and industry. The bibliometric analysis demonstrated the scientific and technological advances that enable the precise control of fluids at a microscopic scale. Major world powers have been developing and allocating resources toward developing this technology through the collaboration of institutions and researchers from various fields who have dedicated themselves to studying the fundamentals of two-phase microchannels to provide new solutions. This review of articles revealed China’s remarkable performance in this research area regarding the number of publications, while the USA exhibited better results in terms of citations. Therefore, it is worth noting that this research area is poised for rapid and extensive progress, with the possibility of a significant increase in the publication of new studies in the coming years.

The versatility of microfluidics results from a combination of sophisticated technologies and tools. Microfluidic channels, separation microchips, microreactors, and micropumps are just a few examples of the components that make up these systems. Lithography and 3D printing, in turn, enable the creation of devices with complex geometries and specific functionalities.

Moreover, the applications of microfluidics are transforming various sectors. In biology and biomedicine, microfluidics enhances disease diagnosis, the development of new drugs, and tissue engineering. In chemistry, it enables chemical reactions on a microscale with greater control and efficiency. In engineering, it finds applications in microelectronics, microdosing systems, and energy production. Additionally, microfluidics has been explored in fields such as biomimetics and sensors, demonstrating its potential for creating nature-inspired systems and susceptible detection devices.

The future of microfluidics is promising, with various emerging fields and applications that transcend the current boundaries of science. Continuous miniaturization, integrating different technologies, and developing new materials are the main challenges that need to be overcome for microfluidics to realize its full potential.
